# Cell Interactions and Patterned Intercalations Shape and Link Epithelial Tubes in *C. elegans*

**DOI:** 10.1371/journal.pgen.1003772

**Published:** 2013-09-05

**Authors:** Jeffrey P. Rasmussen, Jessica L. Feldman, Sowmya Somashekar Reddy, James R. Priess

**Affiliations:** 1 Fred Hutchinson Cancer Research Center, Seattle, Washington, United States of America; 2 Howard Hughes Medical Institute, Chevy Chase, Maryland, United States of America; 3 Molecular and Cellular Biology Program, University of Washington, Seattle, Washington, United States of America; 4 Department of Biology, University of Washington, Seattle, Washington, United States of America; University of California San Diego, United States of America

## Abstract

Many animal organs are composed largely or entirely of polarized epithelial tubes, and the formation of complex organ systems, such as the digestive or vascular systems, requires that separate tubes link with a common polarity. The *Caenorhabditis elegans* digestive tract consists primarily of three interconnected tubes—the pharynx, valve, and intestine—and provides a simple model for understanding the cellular and molecular mechanisms used to form and connect epithelial tubes. Here, we use live imaging and 3D reconstructions of developing cells to examine tube formation. The three tubes develop from a pharynx/valve primordium and a separate intestine primordium. Cells in the pharynx/valve primordium polarize and become wedge-shaped, transforming the primordium into a cylindrical cyst centered on the future lumenal axis. For continuity of the digestive tract, valve cells must have the same, radial axis of apicobasal polarity as adjacent intestinal cells. We show that intestinal cells contribute to valve cell polarity by restricting the distribution of a polarizing cue, laminin. After developing apicobasal polarity, many pharyngeal and valve cells appear to explore their neighborhoods through lateral, actin-rich lamellipodia. For a subset of cells, these lamellipodia precede more extensive intercalations that create the valve. Formation of the valve tube begins when two valve cells become embedded at the left-right boundary of the intestinal primordium. Other valve cells organize symmetrically around these two cells, and wrap partially or completely around the orthogonal, lumenal axis, thus extruding a small valve tube from the larger cyst. We show that the transcription factors DIE-1 and EGL-43/EVI1 regulate cell intercalations and cell fates during valve formation, and that the Notch pathway is required to establish the proper boundary between the pharyngeal and valve tubes.

## Author Summary

Tubes composed of epithelial cells are universal building blocks of animal organs, and complex organs typically contain multiple interconnected tubes, such as in the digestive tract or vascular system. The nematode *Caenorhabditis elegans* provides a simple genetic system to study how tubes form and link. Understanding these events provides insight into basic biology, and can inform engineering strategies for building or repairing cellular tubes. A small tube called the valve connects the two major tubular organs of the nematode digestive tract, the pharynx and intestine. The pharynx and valve form from the same primordium, while the intestine forms from a separate primordium. Cells in each primordium polarize around a central axis, and valve formation involves connecting these axes. Using live imaging, we show that valve cells initially resemble other pharyngeal cells, but undergo additional and extensive intercalations around the lumenal axis, effectively squeezing a small tube from the larger primordium. Valve cells develop the same polarity axis as intestinal cells, and we show that this depends on interactions with the intestinal cells. We show that valve formation involves dynamic changes in the localization of adhesive proteins, and identify transcription factors that play a role in valve cell specification and intercalation.

## Introduction

**Figure 1:**
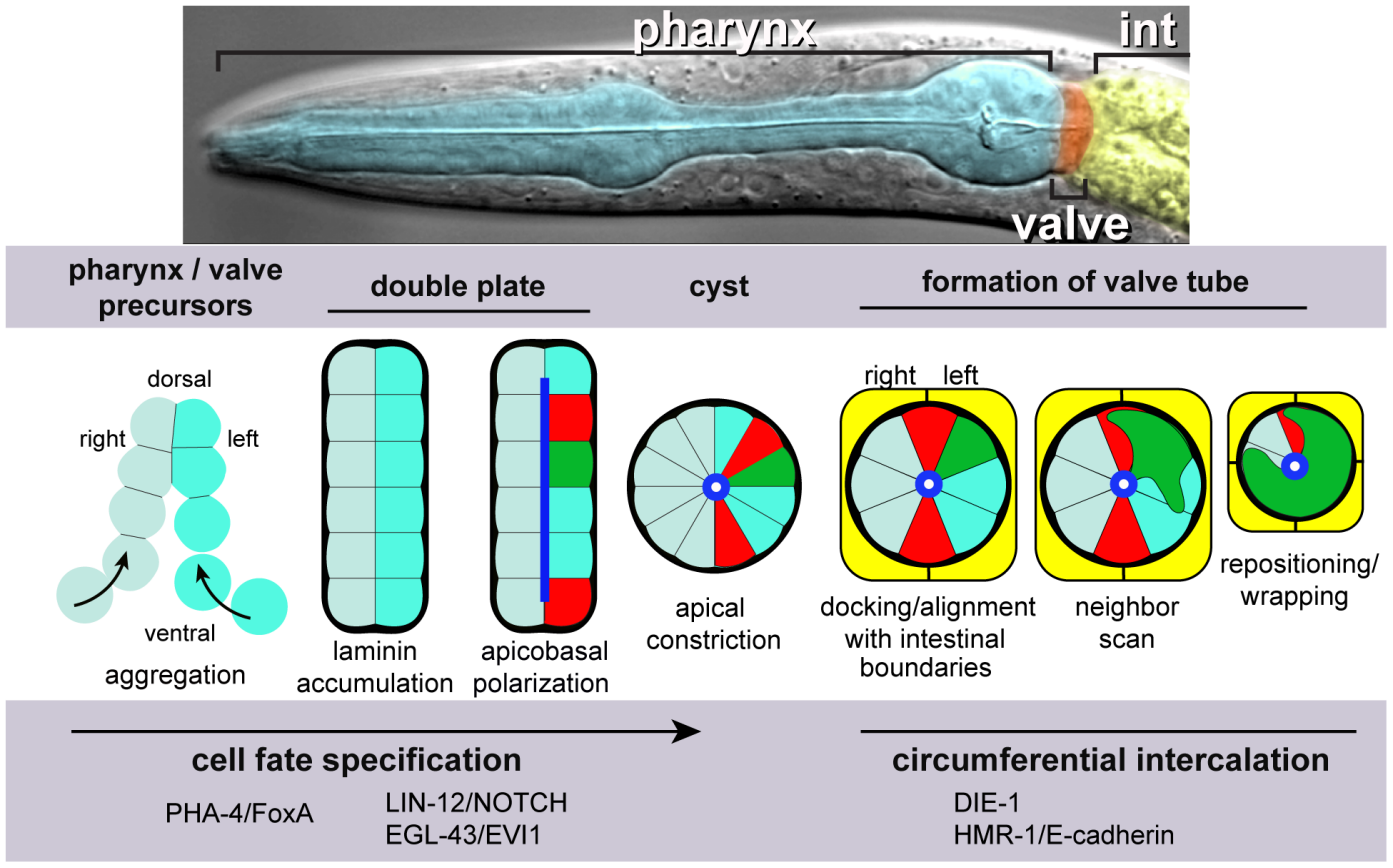
Pharynx/valve morphogenesis. Top, differential interference contrast micrograph of a *C. elegans* larva, false colored to highlight the pharynx, valve and intestine. Bottom, diagram illustrating major stages in the formation of the cyst, and subsequent events as described in this report on the morphogenesis of the valve tube from the cyst; the diagram shows transverse views of all stages. Future pharyngeal and valve cells aggregate into an intermediate structure called the double plate. Laminin (bold black) at the periphery, or basal surface, of the double plate cues the opposite localization of apical proteins (blue), and apical constriction transforms the double plate into a cyst. Two future valve cells (red, called v3D and v3V) dock at the left-right boundary of the intestinal primordium (yellow). Docking begins slightly before, and continues during, apical constriction. The v3 valve cells remain at the left-right boundary until the two intestinal cells divide and form the final, four-cell terminus of the intestine. Valve cells and other cells in the cyst appear to explore their neighbors through actin-rich lamellipodia, and in many cases reposition their cell bodies. pm8 (green) and valve cells wrap partially or completely around the midline, thereby extruding the valve tube from the cyst.

Epithelial tubes are fundamental components of animal organs, and perform many functions such as the transport of liquids, gases or food (reviewed in [1]). Epithelial tubes range in shape from simple cylinders to the branched, convoluted structures of the Drosophila trachea or mammalian kidney (reviewed in [2], [3]), and tube formation can involve extensive remodeling of the constituent cells [4]–[8]. The *C. elegans* digestive tract provides a genetic model system for understanding how epithelial tubes form and remodel. The tract consists primarily of three linked tubes, the pharynx, valve, and intestine (Figure 1). The intestine is a simple, cylindrical tube composed of 20 similar cells that derive from a single early blastomere [9]. The pharyngeal tube is similar in size to the intestine, about 50 microns at hatching, and has only a slightly more complex shape. However, the pharynx is derived from multiple early blastomeres, and contains 80 cells that differentiate into five cell types [9], [10]. The different pharyngeal cell types have diverse shapes, and even cells of the same type have distinct, position-specific morphologies associated with pharyngeal structure and function [10]. The intestine and pharynx form from separate, but adjacent, primordia that polarize at different times in development, apparently using different polarization cues [11], [12]. The valve tube, which contains only six cells, links these larger tubes to form a continuous digestive tract [9], [10].

The pharyngeal primordium includes the future valve cells, and begins as an aggregate of precursor cells that each express the transcription factor PHA-4/FoxA, a key regulator of pharyngeal development (Figure 1) [13]–[15]. The precursors organize into a bilaterally symmetrical array we call the double plate, which resembles two adjacent plates of cells, each plate one cell in thickness (Figure 1) [12], [16]. After cell ingression and division complete the double plate, PAR polarity proteins localize near the junction of the left and right plates; cell membranes at the junction are the future apical surfaces. This localization appears to result from a laminin-dependent cue at the opposite, future basal, surfaces at the perimeter of the double plate. Apical constriction reshapes most cells into wedges and transforms the double plate into a rounded cyst (Figure 1) [12].

How cyst cells remodel into the valve has not been analyzed. However, previous studies suggest that multiple mechanisms contribute to individual pharyngeal cell shapes. Pharyngeal gland cells have long, thin processes that connect to the lumen and secrete mucin-like proteins [10], [17]. The basic gland cell shape appears to result from the cell body migrating away from the site of lumen attachment, spooling out the secretory process in its wake, and gland cell shape is further modified by interactions with surrounding pharyngeal muscles [9], [18]. The longitudinal processes of some pharyngeal neurons may develop from a similar spooling mechanism, as they develop independently of several genes required for axon guidance [19]. In the anterior of the cyst, where pharyngeal cells must ultimately link with epidermal cells to complete the digestive tract, remodeling does not appear to involve either cell migration or cell intercalation [20]. Instead, the anterior cyst cells reorient their apicobasal axes and apical membranes to align with adjacent epithelial cells, thus forming a topologically continuous apical surface [20], [21]. At the posterior of the pharynx, a single donut-shaped cell called pm8 (pharyngeal muscle 8) has the critical role of forming a boundary with the valve. pm8 begins as a dorsal cell in the cyst, but extends a process through ventral cells along a transient tract of laminin. pm8 then autofuses into a toroid, possibly by wrapping around finger-like projections from neighboring cells [22].

Here, we examine how the valve tube originates from the cyst and links with the pharyngeal and intestinal tubes. The terminal pharyngeal cell, pm8, and all the cells that form the valve are located at the posterior of the cyst, initially within a disc of polarized, wedge-shaped cells. The adjacent intestinal cells appear to coordinate valve cell polarity by preventing a polarizing cue, laminin, from accumulating at the posterior surface of the disc. Multiple pharyngeal and valve cells extend lateral, actin-rich lamellipodia between neighboring cells that precede several examples of cell repositioning. The developing valve tube must align with the intestinal tube, and we show that linking cells called v3D and v3V dock at the left-right boundary of the intestinal cells. Other cells organize symmetrically around v3D and v3V, and begin bilaterally symmetrical, highly patterned cell intercalations that eventually reshape the disc into three smaller rings of valve cells. These morphogenetic events involve the transcription factors DIE-1 and EGL-43. The pharynx links to the valve at the boundary between pm8 and the most anterior valve cell, v1. pm8 and v1 have similar origins, occupy symmetrical positions in the cyst, and initiate bilaterally symmetrical wrapping around the lumenal axis such that both cells become toroidal or donut-shaped. During wrapping, however, pm8 intercalates asymmetrically, anterior to v1, and thus joins with other pharyngeal cells. Notch signaling occurs in pm8, but not v1, prior to morphogenesis, and our results suggest that Notch signaling regulates multiple, pharyngeal-specific properties of pm8. Thus, the asymmetry in intercalation order is important because only pm8 has the potential to become a pharyngeal cell.

## Results

### Anatomy and Nomenclature

**Figure 2:**
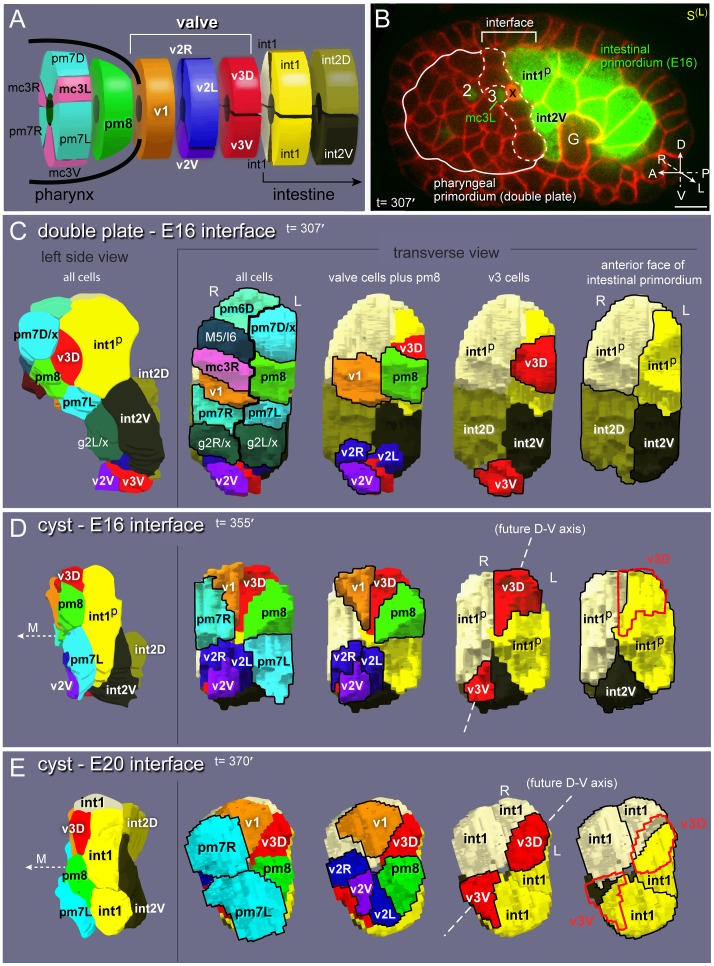
Valve anatomy and origins. (A) Diagram of the valve with neighboring pharyngeal and intestinal cells; see Figure S1 for full cell names. (B) Left sagittal (S^(L)^) optical plane through a live embryo expressing reporters for all plasma membranes (*pie-1*::mCherry::PH(PLC1∂1)) and for intestinal cells (F22B7.9::GFP). The embryo also expresses a *pax-1*::HIS-GFP reporter used for spatial reference; this reporter is expressed in the nuclei of all three groups of marginal cells (numbered 1,2, or 3 throughout this report), pm8 and v1. The solid white line indicates the margins of the double plate. The germ cell precursors (G) and a cell death (x) are visible in this plane. (C) Images from 3D reconstruction of the cell interface between the double plate and E16 intestinal primordia; see also Video S1. Transverse views show all pharyngeal or valve cells that contact intestinal cells, and then selected subsets as labeled. Cells that undergo an additional division are labeled according to their daughters; for example, M5/I6 is the parent of the M5 and I6 neurons, and g2L/x is the parent of the left g2 gland cell and a cell death (x). (D) Reconstruction of the cyst-E16 interface. The pharyngeal and valve cells have acquired wedge shapes through apical constriction. The midline (M) is indicated here and elsewhere by a dashed arrow pointing to the anterior. Note that v3D has shifted dorsally, and begun to center on the left-right boundary between the int1^p^ cells. The intestinal cell int2D has shifted dorsally and behind the left int1^p^ cell, and is not visible in this transverse view; see Video S1. (E) Reconstruction of the cyst-E20 interface. The int1^p^ cells have divided to form the final int1 ring, and both v3D and v3V are centered on the left-right boundaries of the intestinal cells. Bars: (B) 5 microns.

The valve is a short tube of six cells that are organized into three rings, referred to here as rings v1–v3. The first, most anterior ring consists of the single v1 cell, the second contains three v2 cells, and the third contains two v3 cells (Figure 2A). The posterior end of the valve connects with the intestine through a ring of four intestinal cells (the int1 ring); other intestinal cells are organized in sequential rings of two cells each (int2–int9 rings) [9]. All 20 intestinal cells are clonal descendants of an early embryonic blastomere called E; this paper focuses on the E16 and E20 stages of the intestinal primordium, which contain 16 and 20 E descendants, respectively. The anterior end of the valve connects with the pharynx, which consists of myoepithelial cells called pharyngeal muscles (pm), structural cells called marginal cells (mc), epithelial cells, neurons, and gland cells (Figures 2A and S1A) [10]. Most pharyngeal cell types can be subdivided into groups based on region-specific morphological and molecular differences along the anterior-posterior axis (Figures 2A and S1A). Each group contains one to six cells, and cells within a group typically are organized with threefold rotational symmetry around the midline or lumenal axis. Cells can be referenced by a group name such as pm3 (pharyngeal muscle group 3), or by a specific anatomical name such as pm3DL (pm3 Dorsal Left; Figure S1A). For simplicity, we use group names whenever possible, and provide specific names in Figure S1 for reference. Because the *C. elegans* lineage is essentially invariant, most cells at birth have predictable, specific fates within differentiated tissues [9]. In the present study, we found no examples of wild-type fate variability by either direct lineage analysis or by lineage-specific transgene expression. Thus, we refer to each cell at birth by its future, differentiated fate rather than its lineage name. For example, we refer to the cell MSaaapapp simply as pm8.

Tube formation and remodeling were examined by confocal microscopy of live embryos expressing fluorescent reporters. A typical experiment is shown in Figure 2B, and combines reporters for either pharynx-specific or general plasma membranes [*mig-13*::MIG-13::GFP, *pha-4*::GFP::CAAX, or *pie-1*::mCherry::PH(PLC1δ1)] [20], [23], [24], and a marginal cell reporter used for spatial reference [*pax-1*::HIS-GFP] [25]. In addition, we included reporters for intestinal cell membranes and/or nuclei [*end-1*::GFP::CAAX, F22B7.9::GFP] [26], [27]. Optical planes shown are sagittal (S), horizontal (H) or transverse (T) as illustrated in Figure S1A. Cells were identified according to their descent from specific early embryonic blastomeres, and/or by reporter expression and later position within the differentiated pharynx, valve, or intestine.

### Cells Reposition at the Interface between the Pharynx/Valve and Intestinal Primordia

We examined cell contacts as the pharyngeal and valve precursors aggregate into the double plate primordium, and as the double plate transforms into the cyst (Figures 1 and S1B). Many pharyngeal cells maintained contact with their immediate neighbors during the double plate to cyst transformation, consistent with previous observations on nuclear positions [16]. By contrast, cells at the posterior end of the double plate, which include pm8 and all of the valve cells, appeared to change many of their neighbors (Figure S1B). To better visualize these events, we identified and traced contours of all cells at the interface between the pharyngeal/valve primordium and the intestinal primordium, then used the tracings to generate 3D models of the interface cells (Figure 2C–2E, Video S1).

At about 307 minutes in embryogenesis (double plate-E16 stage), the interface contains 19 cells that include the valve cells and diverse pharyngeal cell types (Figure 2C). All of the valve cells and most pharyngeal cells have completed their terminal divisions, and most are cuboidal in shape. The valve cells are split between separate dorsal (v1, v3D) and ventral clusters (v2R, v2L, v2V, v3V). The anterior end of the E16 intestinal primordium contains four cells at this time, but these are not the same four cells that comprise the anterior, int1 ring of the intestine (compare Figure 2C with 2A): The dorsal two intestinal cells at the interface are the parents of the int1 cells that form the int1 ring. We refer to these dorsal cells throughout this report, and for convenience call them the int1^p^ cells (for int1 parents). The ventral two intestinal cells at the interface (int2D and int2V) eventually form the second, or int2, ring of the intestine.

By 355 minutes (cyst-E16 stage), the interface has been reduced to 12 cells (Figure 2D). Most pharyngeal and valve cells have undergone apical constriction, resulting in wedge-shaped cells that surround the midline of the cyst (compare transverse views in Figure 2C and Figure 2D) [12]. The previously separate clusters of valve cells have moved closer together, and this movement occurs as the intervening pharyngeal cells split away from the intestinal cells; the only pharyngeal cells that remain at the interface are muscles (pm8, pm7L, and pm7R). As described further below, the dorsal valve cell v3D becomes positioned diametrically opposite v3V (red cells in Figure 2D), and a line drawn between these cells corresponds to a new, dorsal-ventral axis that prefigures bilaterally symmetrical movements of the flanking cells. Only three intestinal cells remain at the interface because one of the ventral cells (int2D) intercalates behind one of the dorsal cells (the right int1^p^ cell), and both dorsal cells spread ventrally.

By 370 minutes (cyst-E20 stage), the pm7 muscles (pm7R, pm7L) have spread to nearly cover the anterior surfaces of the ventral valve cells, thus separating those valve cells from other pharyngeal cells (Figure 2E). The pm7 muscles have lost most of their direct contact with the intestinal primordium. The two int1^p^ cells have divided, and their four daughters close together ventrally to complete the final int1 ring. The int2 cells (int2D and int2V) have now intercalated behind the int1 cells, essentially wrapping clockwise around the lumenal axis (as viewed from the anterior) to form the second, int2 ring of the intestine. We conclude that the valve cells initially are intermingled with pharyngeal cells, and that both cell types make extensive contacts with intestinal cells. As the cyst forms and begins to remodel, however, only the valve cells and a single pharyngeal cell, pm8, retain contact with the intestinal primordium.

### Docking and Alignment of Valve and Intestinal Cells

The posterior end of the differentiated valve lumen is centered between the v3D and v3V valve cells (Figure 2A). These cells connect with the anterior end of the intestinal lumen, which is centered on the int1 ring at the intersection of the left-right and dorsal-ventral boundaries of the int1 cells (Figure 2A). Because the pharyngeal/valve and intestinal primordia polarize at different times in development, their separate midline axes must be brought into alignment to form a continuous lumenal surface (Figure S2A, S2B) [11], [12]. We found that both v3 cells dock at the left-right boundary of the int1^p^ intestinal cells, and remain there until the dorsal-ventral division of the int1^p^ cells completes the int1 ring.

**Figure 3:**
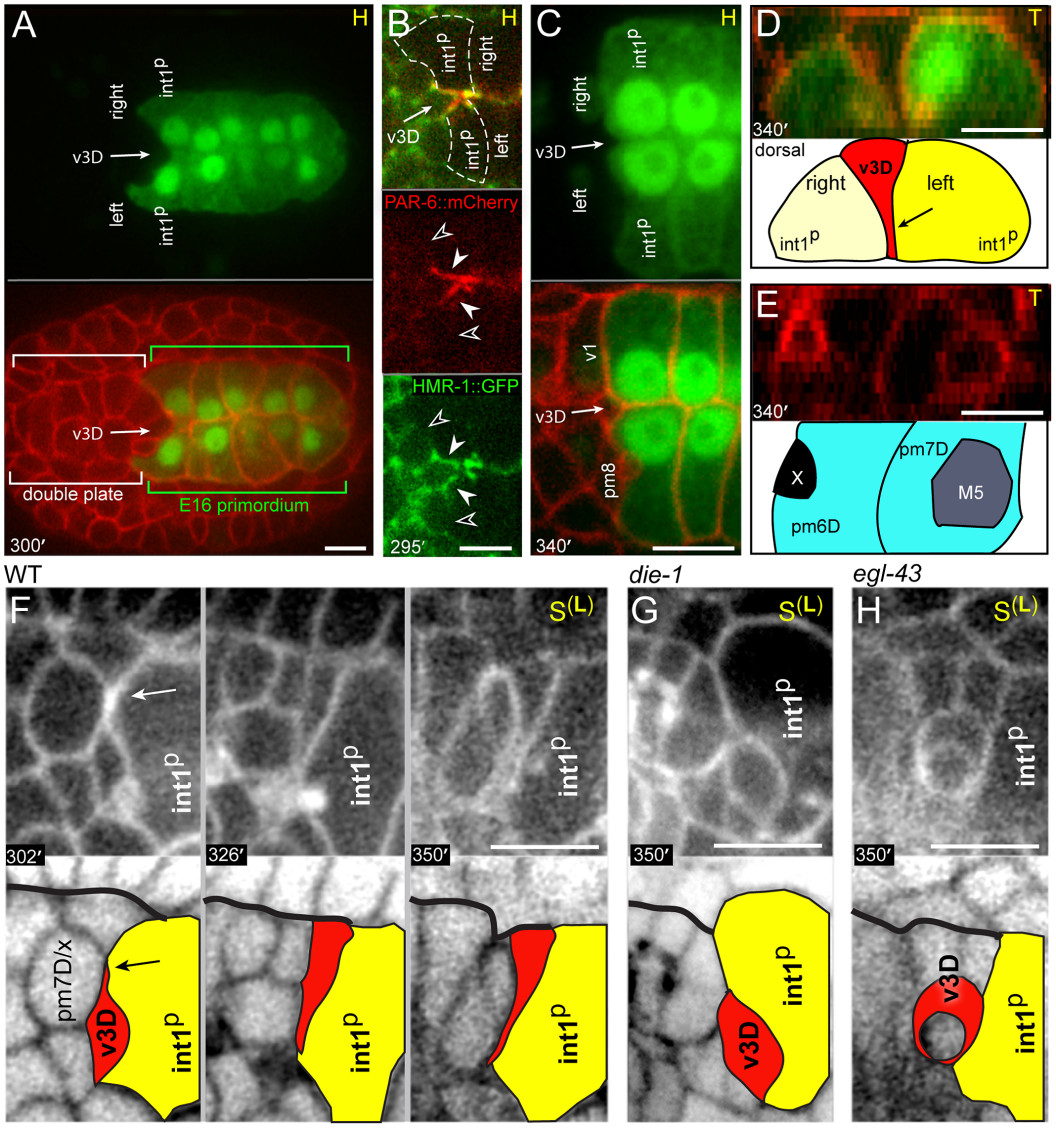
Dorsal alignment of the valve and intestine. (A) Horizontal optical plane near the center of an embryo shortly after the birth of v3D; the top panel shows the E16 primordium and the bottom panel is the same plane showing all cell membranes. Note that v3D is deeply embedded between the left and right anterior intestinal cells (int1^p^ cells). (B) Expression of PAR-6 and HMR-1/E-cadherin at the double plate stage (see Figure S2 for developmental sequence). Closed arrowheads indicate contacts between intestinal cells and v3D, and open arrowheads indicate contacts between intestinal cells and other cyst cells. (C) High magnification through a plane as in panel A, but taken at the late double plate stage. A shallow cleft (arrow) occupied by a process from v3D remains at the left-right boundary between the int1^p^ intestinal cells. (D) Transverse plane showing the wedge-shape of the v3D cell body between the left and right int1^p^ cells. (E) Transverse plane just anterior to the plane shown in panel D, showing the cuboidal shapes of other double plate cells. (F) Time-lapse sequence to 350 minutes showing the intercalation of v3D dorsally across the int1^p^ cells. The arrow indicates a lamellipodium that leads v3D intercalation. The bold black line indicates the dorsal margin of the double plate. (G) v3D in a *die-1(w34)* embryo at 350 minutes. (H) v3D in a *egl-43(zu471)* embryo at 350 minutes; v3D has partially engulfed a cell death. Bars: (A–H) 5 microns.

The parent of v3D creates or occupies a deep pocket between the left and right int1^p^ cells as the latter cells become polarized; after division, the sister of v3D undergoes apoptosis [9] while v3D continues to occupy the pocket (Figure 3A). Reporters for the adhesive protein HMR-1/E-cadherin and the polarity protein PAR-6 become highly enriched where the intestinal cells contact v3D (closed arrowheads, Figure 3B), but not at adjacent contacts with other cyst cells (open arrowheads, Figure 3B; see also S2C). The v3D cell body spreads dorsally during the next 40 minutes, however, a thin extension remains embedded in a small cleft along the left-right boundary between the int1^p^ cells (Figures 3C and S2C). Thus, in transverse view v3D has a wedge shape (Figure 3D) that resembles the shape of later cyst cells, but that contrasts with the shape of its contemporary neighbors in the double plate primordium (Figure 3E). This change in v3D shape appears to be initiated by a dorsal-directed lamellipodium (arrow, Figure 3F) rather than by apical constriction, and begins before other pharyngeal or valve cells show a polarized localization of PAR proteins (Figure S2B).

**Figure 4:**
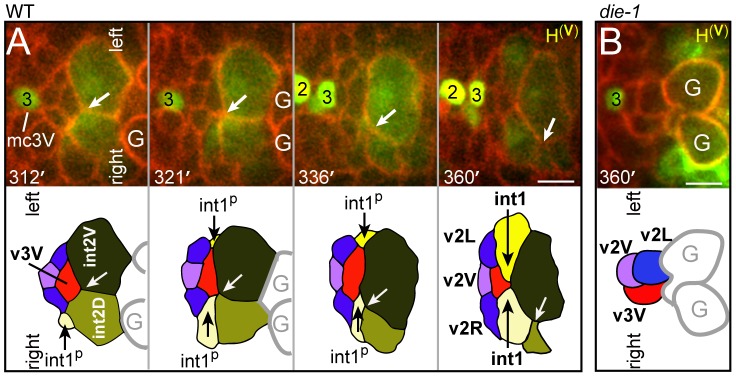
Ventral alignment of the valve and intestine. (A) Time-lapse sequence to 360 minutes showing a horizontal plane through the ventral side of the cyst; all four valve cells in the ventral cluster are visible, and colored as in Figure 2C. Note that v3V does not track the left-right boundary between the int2 cells as that boundary (white arrow) shifts clockwise (down in panel). Processes from the dorsal int1^p^ cells intercalate ventrally to flank v3V, and remain as the int1^p^ cells divide into the four int1 cells. (B) Ventral valve cells in a *die-1(w34)* embryo at 360 minutes. Note that the v2L and v3V cells directly contact the germ cell precursors (G). Bars: (A–B) 2.5 microns.

The v3V cell, which is the ventral counterpart of v3D, initially shows an analogous localization at the left-right boundary between ventral E16 cells (int2D and int2V; white arrow in Figure 4A; see also Figure 2C). However, v3V does not, and cannot, remain at this boundary because int2D and int2V do not contribute to the int1 ring (Figure 4A, Video S1). Instead, the int1^p^ cells spread ventrally until they reach, and stop at, the left and right margins of v3V (Figure 4A; see also Figure 2E). Thus, both v3D and v3V align at the left-right boundary of the int1^p^ cells, near the final position of the intestinal lumen; v3D moves to the intestinal cell boundary, whereas the boundary extends to flank v3V.

### DIE-1 Is Required for the Alignment of the Valve and Intestine

**Figure 5:**
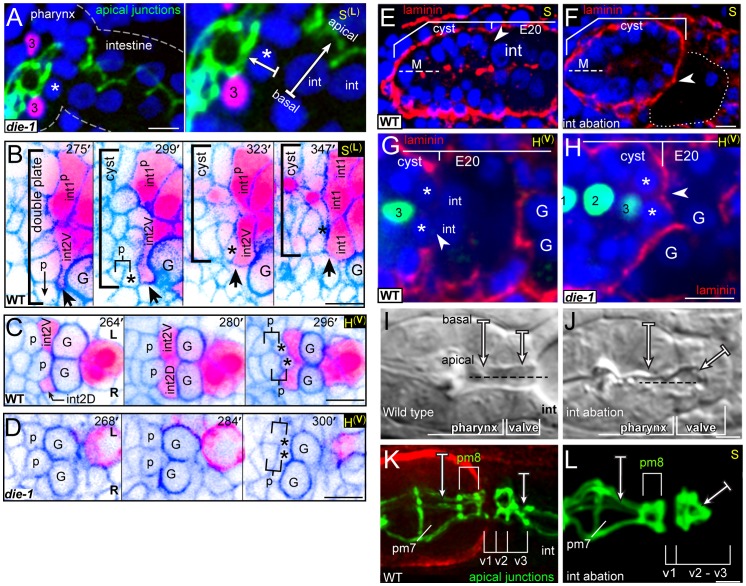
Intestinal cells influence valve cell polarity. (A) Terminal stage *die-1(w34)* mutant embryo. The apical junctions (green, AJM-1) are discontinuous between the pharynx and the intestine, with several nuclei (blue, DAPI) visible in the gap. Compare to the wild-type junctional pattern in panel K. High magnification panel to the right indicates the inferred apicobasal axes of an intestinal cell and a neighboring, presumptive ventral valve cell (asterisk). The valve cell is identified here by its proximity to the ventral mc3 nucleus (#3, magenta; *pax-1*::HIS-GFP). (B) Developmental sequence of the interface between pharyngeal/valve and intestinal cells; reporters are as in Figure 2B, but the colors are inverted to highlight the intestinal cell bodies (magenta). Note extension from int2V (bold arrow) that reaches the parent of v2L (labeled p) at 275 minutes. After the birth of v2L (asterisk; 299 minutes), int2V spreads between the posterior surface of v2L and a germ cell precursor (labeled G). The association of the intestinal cells with v2L persists as cells remodel and the interface (bracket) condenses. (C) Horizontal plane through the ventral side of the double plate and cyst showing the intercalation of the ventral intestinal cells. (D) Image sequence as in panel C but showing a *die-1(w34)* mutant embryo. Note that v2L and v2R (asterisks) remain in contact with the germ cell precursors (G). (E) Laminin (red) surrounds the normal cyst and intestinal primordium, except where the posterior cyst contacts intestinal cells (arrowhead); laminin also appears along the midline (M) at this stage. (F) Image as in panel E after ablating the intestinal precursor (dotted outline). Note that laminin extends across the posterior surface of the cyst (arrowhead). (G) Horizontal plane through the ventral side of a wild-type cyst showing the absence of laminin on the posterior surfaces of v2L and v2R (asterisks), where these cells contact intestinal cells (int). (H) Image of a *die-1(w34)* embryo oriented as in panel G, showing ectopic laminin (arrowhead) across the posterior surfaces of v2L and v2R. (I) Normal pharynx and valve in a newly hatched larva, showing parallel, radial axes of apicobasal polarity of pharyngeal cells and valve cells. (J) Wild-type larva after ablating the intestinal precursor. Note the inferred axis of polarity for the posterior valve cells is oriented to the anterior. (K) Normal apical junctions (AJM-1) and basement membrane (red, UNC-52/Perlecan) in the posterior pharynx and valve. For description of apical junction pattern relative to cell shapes, see [22]. (L) Apical junctions (*dlg-1*::DLG-1::GFP) in a live, wild-type larva after ablating the intestinal precursor as in panel J. Bars: (A–H) 5 microns; (I–L) 2.5 microns.

Previous studies showed that the zinc-finger transcription factor DIE-1 (*d*orsal *i*ntercalation and *e*longation defective-1) is broadly expressed in pharyngeal and intestinal precursors at the late double plate stage, and that *die-1* mutants can have variable gaps in the apical junctions of the digestive tract [28]. We fixed and immunostained *die-1* embryos with an antibody that recognizes a component of apical junctions and found that the gaps in the apical junctions of the digestive tract occurred anterior to the intestine, near the normal positions of valve cells (4/11 *die-1* embryos, Figure 5A). These gaps contained multiple cells and were associated with a misalignment of the pharyngeal and intestinal midlines. In the wild type digestive tract, pharyngeal, valve, and intestinal cells all share a common, radial axis of apicobasal polarity [10], [11]. By contrast, some presumptive ventral valve cells in *die-1* mutants had axes of apicobasal polarity that were not aligned with the adjacent intestinal cells (Figure 5A).

We examined the development of *die-1* mutant embryos to determine the origin of the apparent valve defects. In each of four *die-1* embryos, v3D was born at its normal position in the double plate, but v3D did not extend dorsally along the left-right boundary between the int1^p^ cells (Figure 3G). In one of these embryos, v3D extended an abnormal process between the int1^p^ and int2 cells, but the process later retracted (data not shown). On the ventral side of the cyst, v3V was born at the normal time and place in the *die-1* embryos. However, v3V remained abnormally rounded, and showed little or no contact with the int1^p^ cells (Figure 4B and data not shown). Thus, both v3D and v3V require DIE-1 activity for proper association with the left-right intestinal boundary.

### Intestinal Cells Influence Valve Cell Polarity through DIE-1-Mediated Cell Intercalations

In the course of analyzing *die-1* embryos, we noticed penetrant and abnormal cell contacts between the germ cell precursors and one or more of the ventral valve cells (compare Figure 4B with 4A at 360 minutes). During the wild-type double plate stage, processes from the int2 intestinal cells begin to intercalate between the germ cell precursors and the parents of the valve cells v2L and v2R (each valve cell parent is labeled p in Figure 5B, 5C). By the time v2L and v2R are born (asterisks in Figure 5B, 5C), their posterior surfaces are covered completely by the int2 cells. Shortly thereafter, the additional ventral valve cells (v2V and v3V) intercalate between v2L and v2R, and similarly are separated from the germ cell precursors (Figure 4A and data not shown). The int2 processes cover the posterior surfaces of the ventral valve cells until these contacts are replaced by processes from the int1^p^, and finally int1, cells as described above (Figure 5B). Thus, intestinal cells normally cover the posterior surfaces of the ventral valve cells through all stages of cyst formation and remodeling (brackets, Figure 5B). By contrast, we found that the intestinal cells of *die-1* mutants either failed to spread across the posterior surfaces of the ventral valve cells, or the intestinal cells extended only small processes that later retracted (Figure 5D).

The close association of intestinal cells with the posterior surfaces of valve cells was intriguing for two reasons. First, laminin at the periphery of the double plate appears to orient the apicobasal polarity of many or all pharyngeal cells [12]. Second, despite accumulating at other peripheral surfaces of the double plate and cyst, laminin appears to be excluded from the posterior surface (arrowhead, Figure 5E). We identified the valve cells v2L and v2R in fixed and immunostained wild-type embryos at the double plate and cyst stages (asterisks in Figure 5G), and confirmed that laminin is not detectable at the posterior surfaces of these cells (arrowhead, Figure 5G). To determine whether contact with the intestinal primordium prevents laminin accumulation at the posterior of the double plate and cyst, we used a laser microbeam to kill the intestinal precursor in 12-cell, wild-type embryos. Each of 18 embryos that developed without an intestinal primordium showed ectopic laminin accumulation on part or all of the posterior surface of the double plate and cyst (arrowhead, Figure 5F). Similarly, we found that unablated *die-1* mutants embryos had ectopic laminin on the posterior surfaces of v2L, v2R, and neighboring cells that fail to contact intestinal cells (arrowhead, Figure 5H).

Ablated wild-type embryos that were allowed to develop to terminal stages all developed a relatively normal pharynx, with a basement membrane delimiting the pharynx from the valve (n = 20; Figure 5I, 5J). Pharyngeal cells in the ablated embryos appeared to have the normal, radial axis of apicobasal polarity, as did the most anterior valve cells (Figure 5I–5L); indeed, two of the embryos hatched and ingested material (data not shown). However, the posterior valve cells formed an abnormal, ball-like structure surrounding a truncated lumen (Figure 5J, 5L). The apical surfaces of the most posterior valve cells were oriented anteriorly, indicating that they have a longitudinal or oblique axis of apicobasal polarity instead of the normal, radial axis (Figure 5J, 5L). These results suggest that contact with the intestinal primordium normally contributes to valve cell polarity by preventing a polarizing cue, laminin, from accumulating on the posterior surfaces of valve cells. Moreover, these results suggest that the apical junction and polarity defects in *die-1* mutant embryos stem from earlier defects in intestinal cell intercalation.

### Cyst Cells Scan Their Neighbors via Lateral, Actin-Rich Lamellipodia

**Figure 6:**
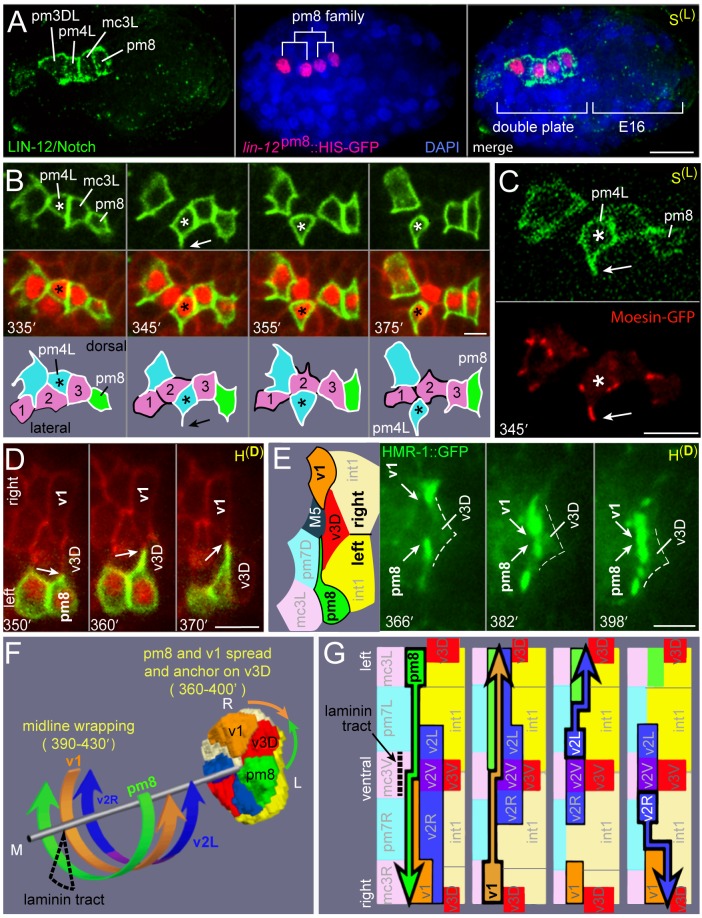
Cyst cells probe their neighbors. (A) Embryo at the double plate stage showing LIN-12/Notch immunostaining (green) and simultaneous expression of a nuclear-localized transcriptional reporter for the pm8 family (red, *lin-12* ^pm8^::HIS-GFP). (B) Time-lapse sequence of the pm8 family at the cyst stage; see also Video S2. The pm8 family members express membrane and nuclear reporters (green, *lin-12* ^pm8^::mCherry::CAAX; red, *lin-12* ^pm8^::HIS-GFP). All pharyngeal/valve cells express an additional membrane reporter (red, *pha-4*::GFP::CAAX). Sequence shows several dynamic lamellipodia, including one from pm4L (arrow) that appears to lead it through a row of marginal cells (1 = mc1DL, 2 = mc2DL, 3 = mc3DL). (C) pm8 family members expressing the above membrane reporter (green) plus a reporter for filamentous actin (red, *lin-12* ^pm8^::GFP::dMoeABD). Note concentration of actin at tip of pm4 lamellipodium (arrow) and other lamellipodia. (D) Horizontal plane through the dorsal roof of the cyst, showing lamellipodia extending from pm8 and v1 and covering the anterior face of v3D; cells in this panel are identified in Figure S1C. (E) Image sequence similar to panel D, but showing HMR-1/E-cadherin expression (green, HMR-1::GFP). (F) Cartoon summarizing circumferential intercalations in the cyst. (G) Diagrams of the posterior cyst shown flayed along the dorsal margin and flattened. Superimposed on each diagram is the intercalation path of the cell outlined in bold. Bars: (A) 10 microns, (B–E) 2.5 microns.

A comparison of interface cells at the late cyst stage (Figure 2E) with their final positions and shapes (Figure 2A) indicates that extensive remodeling must occur after the v3 cells dock with the intestine. To visualize the behaviors of individual cells in detail, we wanted to develop a reporter that was expressed in a subset of the interface cells. Previous studies showed that LIN-12/Notch is expressed in a large number of cells throughout embryogenesis, including pm8 and its immediate relatives near the time of their birth in the double plate primordium (Figure 6A) [22]. We identified an enhancer element (*lin-12* ^pm8^) within a *lin-12* intron that can drive transgene expression in pm8 and its relatives, but not in other cells that normally express LIN-12 (Figure 6A; Materials and Methods). The pm8 relatives include two muscles (pm3DL and pm4L) and one marginal cell (mc3L); for convenience we refer to pm8 and these three cells as the pm8 family.

We used the *lin-12* ^pm8^ enhancer to construct membrane-localized and nuclear-localized reporters, and used these in conjunction with non-specific membrane reporters to examine the development of the pm8 family. We found that all cells in the pm8 family developed dynamic lamellipodia that appeared to probe neighboring cells during the cyst stage (Figure 6B, Video S2). We constructed a strain where the pm8 family expresses a reporter for plasma membranes plus a second reporter with the actin-binding domain of moesin fused to GFP [29] (Figure 6C). In time-lapse movies, moesin-GFP was strongly enriched at leading edges of the lamellipodia, suggesting that they are rich in filamentous actin (arrow, Figure 6C). Simultaneous imaging from horizontal and transverse planes showed that the lamellipodia emerge from sub-basal, lateral surfaces of the wedge-shaped cyst cells, and that the lamellipodia usually extend across one, or occasionally two, neighboring cells (Figure 6B, Video S2). While most of the lamellipodia appear transient and variable, several of the longer-lived lamellipodia are highly reproducible and precede cell repositioning. For example, in normal development pm4L is a lateral muscle, but its sister is a dorsal muscle separated from pm4L by a longitudinal row of marginal cells (Figure S1A). In live imaging, we found that these sisters initially are on the same, dorsal side of the marginal cell row. However, pm4L develops a lamellipodium that breaches the marginal cell row, and the bulk pm4L cytoplasm follows the lamellipodium into the lateral muscle group (Figure 6B). Importantly, pm4L maintains its basic wedge-shaped appearance and association with the midline throughout the repositioning, such that the apparent circumferential migration is essentially a rotation of the pm4L cell body around the midline (Videos S2 and S3).

Another prominent lamellipodium extends from the right side of pm8, beginning at about 350 minutes (arrow, Figure 6D). As the lamellipodium crosses the anterior face of v3D, it meets a mirror-image lamellipodium extending from the left side of v1 (Figures 6D and S1C). The pm8 and v1 processes remain, without crossing, on the anterior face of v3D for at least 45 minutes, during most of the remodeling events described below. We found that HMR-1/E-cadherin::GFP becomes highly enriched in or near the tips of the pm8 and v1 lamellipodia as they converge, suggesting that E-cadherin contributes to the stability of these cell contacts (Figure 6E).

### pm8 Becomes a Toroid by Wrapping around the Midline

The above results show that after v3D docks at the left-right boundary between the intestinal cells, the flanking pm8 and v1 cells come together, and possibly anchor, on the face of v3D. At this stage, pm8 and the valve cells are discontinuous, wedge-shaped cells at various positions around the midline of the cyst (Figure 6F). For example, the valve cells that later form the v2 ring (v2R, v2V, and v2L) are all on the ventral side of the midline. At about 350 minutes, the int1^p^ cells divide to generate the final int1 ring, thus defining the position of the intestinal lumen. Shortly after the int1^p^ division, pm8 and the valve cells begin large-scale intercalations that eventually encircle the midline. The general pattern of the intercalations is illustrated in Figure 6F, with specific intercalation paths diagrammed in Figure 6G and described below.

**Figure 7:**
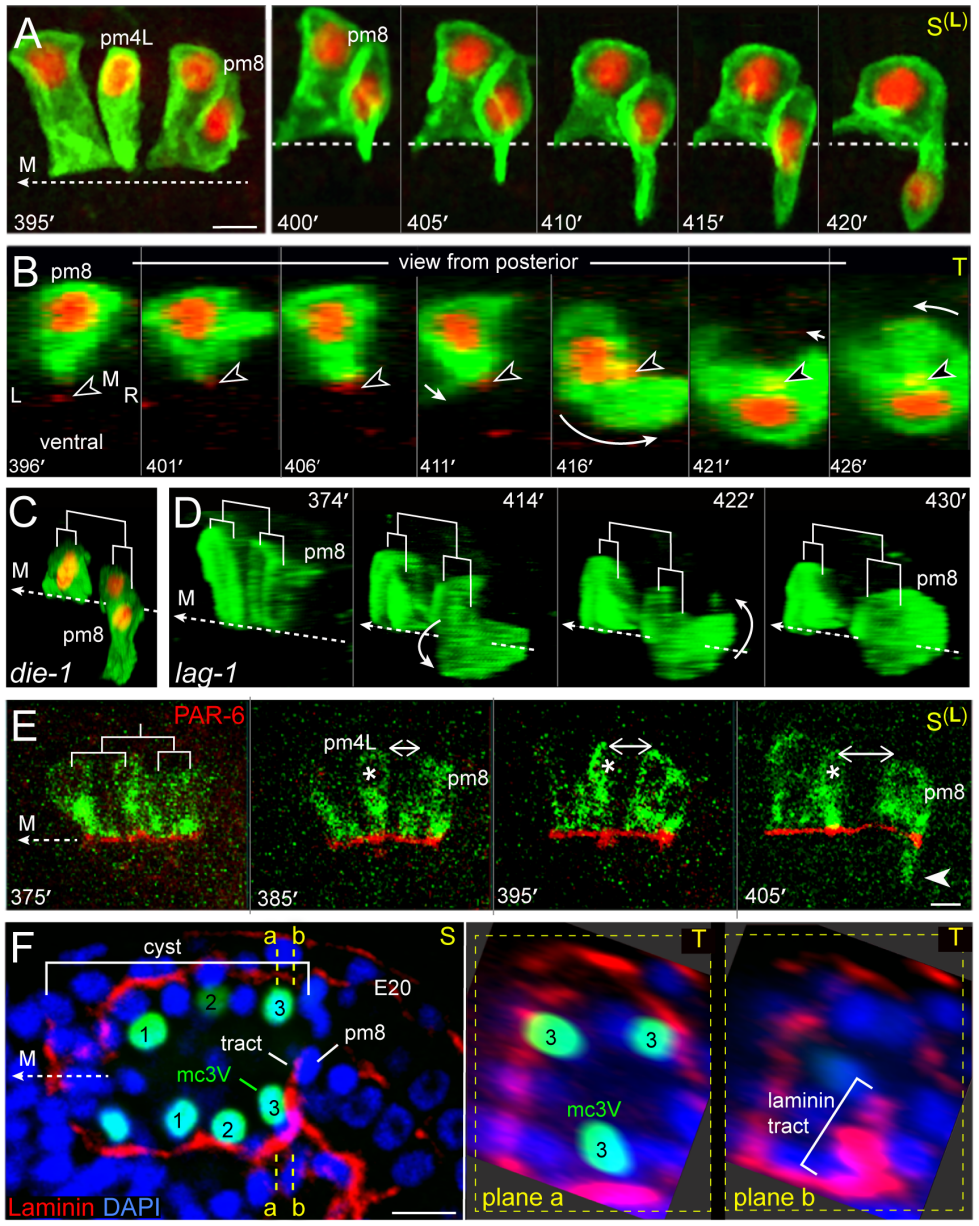
pm8 wraps around the midline. (A) Left sagittal plane showing the pm8 family; cells express nuclear and membrane reporters as in Figure 6B. The right panel shows a time-lapse sequence of pm8 and its sister as pm8 extends ventrally; see also Video S3. (B) Transverse view of pm8 development. This embryo expresses an additional marker (arrowhead) for the midline (red, *nmy-2*::NMY-2::GFP). (C, D) Surface renderings of the pm8 family as shown for the wild-type embryo in Video S3, and rotated to show cell shapes. (C) *die-1(w34)* mutant embryo at about 450 minutes. pm8 has extended ventrally, but the pm8 nucleus remains near the midline and pm8 has not completed wrapping. (D) Image sequence of a *lag-1(q385)* mutant embryo, showing apparently normal pm8 wrapping. (E) pm8 family members expressing a reporter for PAR-6 (red, *lin-12* ^pm8^::PAR-6::GFP) and for cell bodies (green, *lin-12* ^pm8^::SAS-5::mCherry). Note that PAR-6 remains apical as pm4L (asterisk) rotates away from other family members (double-headed arrow; see also Figure 6B), and pm8 begins to wrap (arrowhead). (F) Image of the cyst as the presumptive pm8 nucleus begins to travel ventrally in close association with a tract of laminin. The right panels show transverse planes through positions a and b, and show that the laminin tract has a wedge shape centered on the mc3V nucleus. Bars: (A,E,F) 5 microns.

Between 400 and 410 minutes, pm8 extends a ventral-directed lamellipodium that is followed by the pm8 nucleus (Figure 7A). In transverse view, the lamellipodium sweeps down the left side of the cyst, across the ventral side, and up the right side to close back on itself (Figures 7A, 7B and S3A, S3B, Video S3). This live imaging provides direct confirmation for our previous model that pm8 becomes a toroid or donut-shaped cell by circumferential wrapping [22]. The wrapping requires that pm8 intercalates between pairs of neighboring cells (Figure 6G), and we found that intercalation was defective in *die-1* mutants: pm8 extends a ventral process and shows limited nuclear migration into the process in *die-1* mutants, but pm8 fails to complete wrapping (Figure 7C). Thus, *die-1* mutants appear defective in several different examples of cell intercalation during cyst remodeling.

Because the remodeling of cells in the anterior of the cyst is associated with a re-orientation of the apicobasal axis (see Introduction), we wanted to examine whether remodeling in the posterior cyst might involve changes in cell polarity. At and after the cyst stage, PAR-6 appears to be distributed continuously along the midline of the cyst, suggesting that PAR-6 remains at the apical surfaces of at least some posterior cells during remodeling (Figure S2A and data not shown). We then used the *lin-12* ^pm8^ enhancer to examine PAR-6::GFP expression specifically within the pm8 family. The pm8 family members localized PAR-6::GFP to their midline-facing, apical surfaces during the double plate to cyst transformation, similar to other pharyngeal cells. PAR-6::GFP remained apical throughout the morphogenesis of the pm8 family members, including during the rotational repositioning of pm4L away from the other family members (double-headed arrow in Figure 7E) and the ventral wrapping of pm8 (arrowhead in Figure 7E). Thus, cyst cells appear to maintain their radial axis of apicobasal polarity while remodeling their orthogonal, lateral surfaces.

Previous studies showed that a novel, radially oriented tract of laminin develops within the posterior cyst just before pm8 begins to change shape (Figure 7F): The ventral extension of pm8 occurs in close proximity to this tract, and laminin function is required for normal pm8 morphogenesis (Figure 7F) [22]. With transverse optical sectioning, we found that the laminin tract has a well-defined wedge shape that closely approximates the shape of the adjacent marginal cell, mc3V (Figure 7F and data not shown). This suggests that the pm8 lamellipodium encounters the tract only after it begins to probe between ventral cells, and raises the possibility that the different behaviors of the various pm8 lamellipodia result from the different environments they encounter.

### Extrusion of the Valve Tube by E-cadherin-Mediated, Circumferential Intercalation

About 10–15 minutes after pm8 begins to extend ventrally on the left side of the cyst, v1 develops a symmetrical, ventral-directed lamellipodium on the right side of the cyst (Figures 6F, 6G and S3C). The pm8 and v1 lamellipodia meet and pass dorsal-directed lamellipodia extending from v2L and v2R, respectively (Figure S3C, S3D, Video S4). The v2L and v2R lamellipodia precede their nuclei and bulk cytoplasm, shifting both cells bodies to the dorsal side. Similar to the pm8 and v1 intercalations, v2L and v2R appear to follow invariant trajectories through the cyst. For example, the v2R lamellipodium initially spreads dorsally between an int1^p^ daughter (one of the ventral int1 cells) and the v1 lamellipodium or cell body (n = 9 embryos; Figure 6G and S3C). The timing of the v2L and v2R intercalations are highly reproducible with respect to the timing of the int1^p^ divisions: Shortly after the left (or right) int1^p^ cell divides into a dorsal-ventral pair of int1 cells, v2L (or v2R) begins spreading on the adjacent, ventral int1 cell. The migrating v2L (or v2R) nucleus reaches the dorsal-ventral boundary between the int1 cells at 48+/−6 minutes (n = 5 embryos; Figure S3C, S3D). This reproducibility is intriguing because the absolute division times of the int1^p^ cells can vary by up to 20 minutes in different embryos, and the division times of the left and right int1^p^ cells can differ by up to 15 minutes within the same embryo (n = 18 embryos). Thus, we speculate that the int1^p^ divisions might trigger valve cell intercalation, for example by modulating adhesion to the intestinal cell surface.

**Figure 8:**
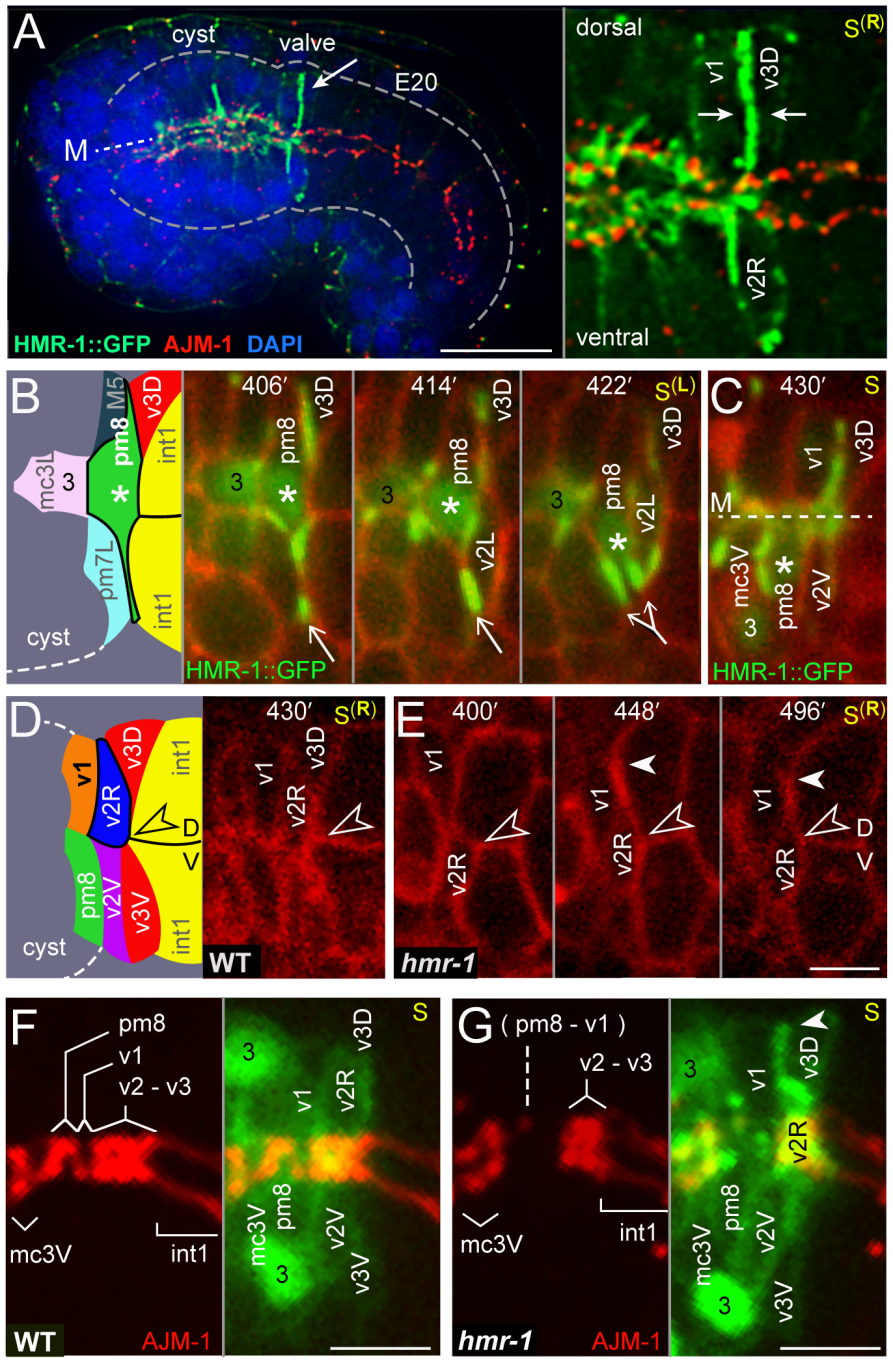
HMR-1/E-cadherin expression and circumferential intercalations. (A) Low magnification of an immunostained embryo showing HMR-1::GFP (green) and the apical junction protein AJM-1 (red). Note the high level of HMR-1 in the region of the developing valve compared to the level in most other embryonic cells. The right panel is a high magnification of the valve region showing HMR-1 enrichment at the boundary between v1 and v3D, and around the v2R cell body. (B) Time-lapse images of a live embryo showing HMR-1::GFP on the left side of the cyst as pm8 moves ventrally; the pm8 nucleus is indicated by an asterisk. Note that HMR-1 associated with the ventral lamellipodium from pm8 (arrow) appears to split as the pm8 nucleus moves toward and into the lamellipodium. (C) Lower, sagittal focal plane of the same embryo as in panel B at 430 minutes. The pm8 nucleus has moved further ventral, and is now adjacent to mc3V (the mc3V nucleus is labeled 3). The line of HMR-1 between pm8 and mc3V is in the same position as the laminin tract shown in Figure 7F. (D) Right sagittal focal plane at 430 minutes of the same embryo shown in panels B and C; the HMR-1::GFP signal has been removed to show cell shapes. v2R has intercalated dorsally, with its bulk cell body past the dorsal-ventral boundary (open arrowhead) between the int1 intestinal cells. (E) Time-lapse of a *hmr-1(zu389)* mutant embryo. The v2R nucleus and most of the cell body remain below the dorsal-ventral int1 boundary through 496 minutes, although the v2R lamellipodium (closed arrowhead) has extended dorsally. (F,G) Apical junctions (red, AJM-1) in a wild-type embryo (F) and a *hmr-1(zu389)* mutant embryo (G), taken at about 440 minutes. For cell identification, the panels to the right in each figure show an image of cell bodies and/or nuclei (green) taken 1.5 microns to the left of the midline and superimposed on the apical junction image. At the stage shown, pm8 and v1 have wrapped around the midline, but not undergone autofusion into donuts; see [22] for detailed description of junctions at this stage. The apical junctions in the *hmr-1* mutants are abnormal, with a large gap in the region of the pm8, v1, and the v2 cells. Similar to the *hmr-1* embryo in panel E, the v2R cell has failed to move dorsally, although its lamellipodium (arrowhead) has intercalated between v1 and v3D. Bars: (A) 10 microns; (B–G) 2.5 microns.

Because E-cadherin has been shown to regulate intercalations in other systems [30], [31], we examined HMR-1/E-cadherin localization during valve cell intercalation. We found that embryos expressing a rescuing HMR-1::GFP transgene [32] showed a striking enrichment of HMR-1 along lateral membranes at the posterior of the cyst during valve formation (arrow, Figure 8A). This localization contrasts with that in intestinal cells and most other cyst cells, where HMR-1/E-cadherin is predominantly at the midline or apical surface in association with apical junction proteins such as AJM-1 (Figure 8A; see also Figure S2B). We identified the dorsal lines of HMR-1 as the boundaries between v3D and either v1 or pm8, as described above for earlier embryos (Figure 6E). This reproducible, and apparently persistent, zone of expression contrasted with variable localization of HMR-1/E-cadherin in the ventral cyst. In live embryos, we found that HMR-1::GFP localization in the ventral cyst is dynamic, and concentrated particularly around pm8 and the subset of valve cells that undergo nuclear migration (v2L and v2R). For example, HMR-1::GFP is enriched in or by the ventral lamellipodium from pm8, initially appearing as a single line of fluorescence (406 minutes, arrow in Figure 8B). The line of HMR-1::GFP then bifurcates as the pm8 nucleus and bulk cytoplasm shift ventrally (422 minutes, Figure 8B). HMR-1::GFP persists at high levels as the pm8 nucleus continues moving to the ventral interior of the cyst, where it is enriched between pm8 and mc3V near the same location as the laminin tract (430 minutes, Figure 8C). Similarly, high levels of HMR-1::GFP flank v2L as the v2L nucleus and cytoplasm move dorsally, but little or no HMR-1::GFP is associated with v2V, which stays largely in place (Figure S3E).

HMR-1/E-cadherin is expressed both maternally and embryonically; previous studies showed that embryos lacking embryonic expression appear to have relatively normal assembly of most tissues and organs, but fail in hypodermis (skin)-mediated processes such as ventral closure and body elongation [33], [34]. We found that *hmr-1* mutant embryos appeared to initiate all of the above intercalation movements, but had defects in nuclear migration and transfer of bulk cytoplasm. For example, the migrating v2R nucleus reached the dorsal-ventral boundary between int1 cells about 48 minutes after the int1^p^ division, similar to wild-type embryos described above. In wild-type embryos, the v2R nucleus and bulk cell body continue to travel dorsally, and pass beyond the dorsal-ventral boundary in an additional 8+/−1 minutes (n = 5 embryos; Figure 8D). However, in *hmr-1* embryos, the v2R nucleus and bulk cell body appeared stalled at the dorsal-ventral boundary for at least 1 hour, although the v2R lamellipodium could extend further dorsal between the valve cells v1 and v3D (arrowheads, Figure 8E; n = 3 embryos). At terminal stages, *hmr-1* embryos showed defects in the apical junctions in the valve, including apparent gaps within, or anterior to, the v2 cells (Figure 8F, 8G). In summary, valve cells create a tube by circumferential intercalation, or wrapping, around the midline. These movements condense the radius of the posterior cyst symmetrically around the midline while extending the longitudinal axis, effectively extruding the small, valve tube from the larger cyst.

### Notch Regulates Post-Morphogenetic Gene Expression in pm8

**Figure 9:**
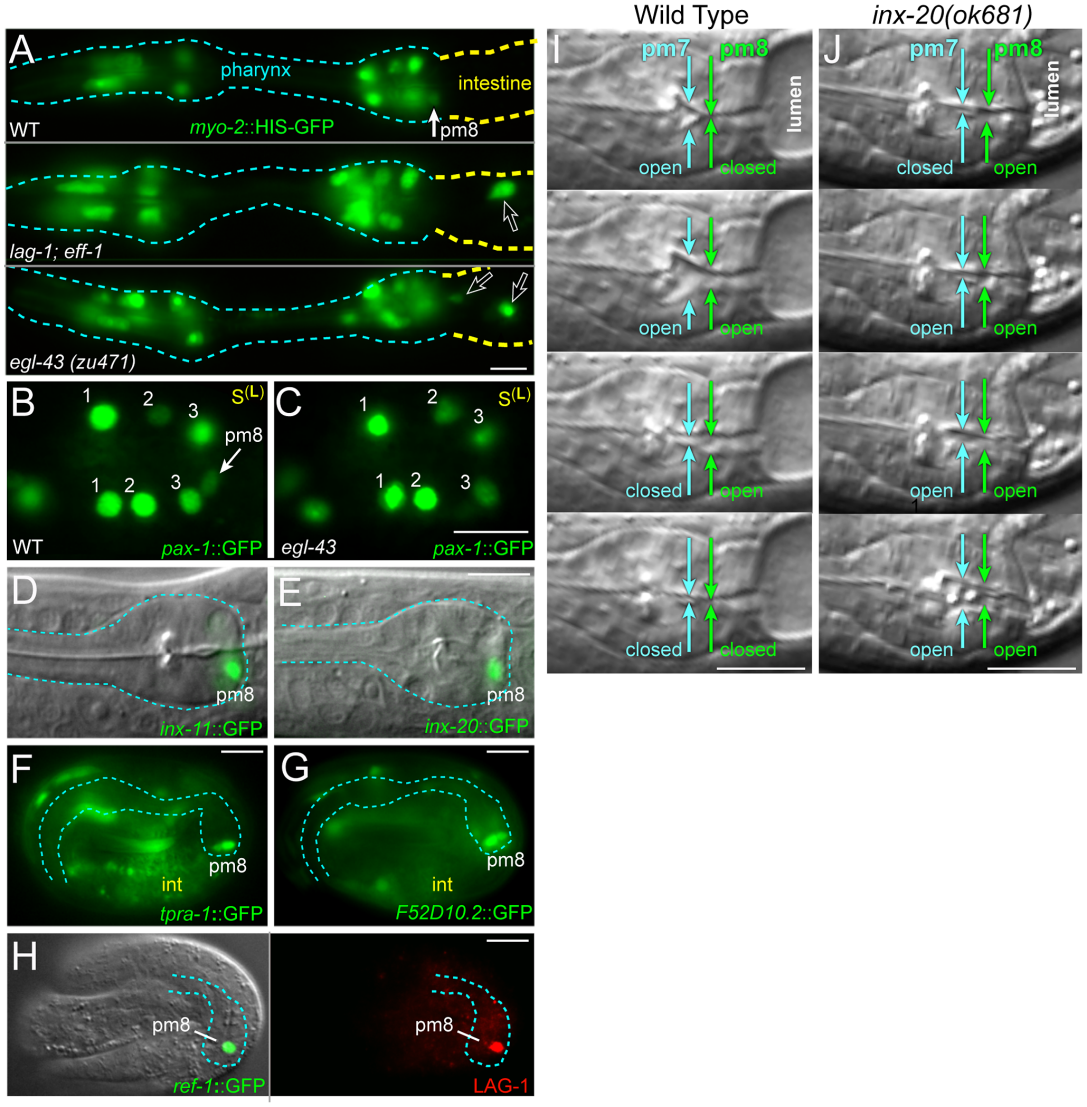
Notch-related valve defects and target gene expression. (A) Newly hatched wild-type, *lag-1(q385); eff-1(ok1021)* and *egl-43(zu471)* mutant larvae expressing a reporter for pharyngeal muscle nuclei (green, *myo-2*::HIS-GFP). Open arrows point to nuclei mispositioned posteriorly between intestinal or valve cells. (B) Wild-type cyst oriented as in Figure 7F, showing *pax-1*::HIS-GFP expression in marginal cell nuclei (#1–3) and in pm8. (C) *egl-43(zu471)* mutant cyst, staged and oriented as in panel B; note the lack of *pax-1*::HIS-GFP at the normal position of pm8. (D–G) Notch-regulated target gene expression in larvae (D and E) and in late embryos as listed; see also Figure S4. (H) Late-stage, wild-type embryo as in panels F and G, immunostained to identify pm8 (green, *ref-1* ^1.8 kb^::REF-1::GFP) and showing LAG-1 (red) in the pm8 nucleus. Note that LAG-1 is absent from other embryonic nuclei, although most or all nuclei express LAG-1 in earlier development (data not shown). (I,J) Pharyngeal pumping sequence in a wild-type L1 larva (I) and an *inx-20(ok681)* (J) mutant larva; see also Video S5. Bars: (A–J) 5 microns.

Larvae that are defective in the LIN-12/Notch signaling pathway have severe defects in the structure of the pm8/valve boundary, although the Notch pathway appears to be activated only in pm8 [22]. At least part of the role of the Notch pathway is to prevent pm8 from fusing with the adjacent v1 cell: In normal development, the toroidal or donut shapes of pm8 and v1 require that each cell undergoes autofusion while avoiding cross-fusion. Notch signaling in pm8 accomplishes this by activating expression of the pm8-specific fusogen, AFF-1, while repressing the expression of the v1-specific fusogen, EFF-1. LAG-1 is the transcriptional effector of the LIN-12/Notch pathway, and in *lag-1* mutants pm8 can express EFF-1 and cross-fuse with v1 [22]. If preventing the expression of EFF-1 were the sole function of Notch signaling, *lag-1; eff-1* double mutants might be expected to have a normal boundary, similar to *eff-1* single mutants. Instead, we found that most *lag-1; eff-1* larvae had boundary defects, with posterior mispositioning of one or more pharyngeal nuclei between valve or intestinal cells (n = 20/32 embryos; open arrows in Figure 9A). A similar mispositioning of pharyngeal nuclei has been described for mutations affecting INA-1/alpha-integrin [22], but is never observed in wild-type larvae (n = 0/100), and only rarely observed in *eff-1(ok1021)* mutant larvae (n = 2/74) or *eff-1(ok1021) aff-1(tm2214)* double mutant larvae (n = 2/20).

To better understand the role of Notch signaling in pm8 and the valve, we made live recordings of *lag-1* embryogenesis using membrane and nuclear reporters for the pm8 family. We found that the timing and pattern of pm8 morphogenesis appeared essentially identical to that of wild-type embryos, with pm8 wrapping around the midline (Figure 7D, compare with wild-type Video S3). We next used a computational approach to search for target genes that might be regulated by Notch signaling in pm8. Most, if not all, of the numerous Notch interactions that occur in embryogenesis regulate expression of the *ref-1* family of transcriptional repressors [35], [36]. However, Notch signaling activates expression of the transcription factor CEH-24 specifically in pm8, suggesting that additional pm8-specific targets might exist [22]. We identified 256 genes that have (1) predicted LAG-1 binding sites (RTGGGAA) in their upstream sequences, and (2) conservation of the LAG-1 sites in at least 3/5 sequenced *Caenorhabditis* genomes (see Materials and Methods; Table S1). Thirteen of these genes have been described in the literature and/or public database annotations as having pharyngeal or pharyngeal muscle expression, and pm8 expression has been noted for five genes in addition to *ref-1* and *ceh-24* [25], [37]–[39]. We created reporters for three of these genes (*pax-1*, *inx-11*, and *inx-20*; Figure 9B, 9D, 9E) and found that mutation of the conserved LAG-1 site to RAGGCAA in each reporter abrogated pm8 expression (Figure S4A–S4C). We further showed that a DNA sequence including the *inx-11* LAG-1 site, but not a mutated version, was able to compete for LAG-1 binding in vitro (Figure S4E). We noticed that *inx-11* and *pax-1* both contained predicted and conserved PHA-4/FoxA binding sites (TRTTKRY) [40] near the LAG-1 site, and found that mutations in the PHA-4/FoxA sites also abrogated pm8 expression (Figure S4A, S4D). 60 of the genes with predicted LAG-1 binding sites had one or more predicted and conserved PHA-4/FoxA sites within 110 bp of the LAG-1 site. Of seven of these genes tested, three showed pm8 expression (*tpra-1*, *F52D10.2*, *F52E4.5*; Figure 9F, 9G and data not shown), and for each of two genes tested the expression was Notch dependent (Figure S4A). These results indicate that the presence of combined, conserved LAG-1 and PHA-4/FoxA sites is a predictor of Notch-activated expression in pm8.

Previous studies showed that Notch-regulated gene expression can be detected within 25 minutes after a Notch-expressing cell contacts a ligand-expressing cell [35]. Because Notch signaling is activated shortly after the birth of pm8 and before morphogenesis, we anticipated that Notch targets would be expressed during pm8 morphogenesis [22]. However, most of the Notch targets examined were expressed only late in embryogenesis, long after pm8 completes wrapping and the LIN-12/Notch protein is no longer detectable by immunostaining (Figure 9D–9G and data not shown). Although we do not understand the basis for the delayed onset of Notch target gene expression, we found that the LAG-1 protein persists at high levels in the pm8 nucleus throughout embryogenesis, several hours after LAG-1 is no longer detectable in any other nucleus (Figure 9H).

We did not observe pharynx/valve defects in mutants defective in the Notch targets *ref-1*, *ceh-24*, *pax-1*, *inx-11*, or *inx-20* (data not shown), but observed pharyngeal pumping defects in *inx-20* mutants. In wild-type larvae, the coordinated contraction of pharyngeal muscles propels food particles posteriorly into the intestine [41], and we found that pm8 normally contracts (opens) only after pm7 contracts (Figure 9I, Video S5). In *inx-20* larvae, we found that the contractions of pm8 and pm7 were not coordinated; for example, pm8 could open before pm7, apparently causing the anterior regurgitation of food particles from the valve or intestine into the pharynx (Figure 9J, Video S5). In summary, Notch has a role in establishing or maintaining the pharynx/valve boundary beyond the regulation of fusogen expression, but does not appear critical for pm8 to complete its basic morphogenetic program of wrapping around the midline. Our identification and analysis of Notch-regulated targets in pm8 suggests that most of these targets do not function in morphogenesis, and instead function in the late, pharyngeal or muscle-specific differentiation of pm8.

### EGL-43/EVI1 Regulates LIN-12/Notch Expression and Functions in pm8 and v3D Development

**Figure 10:**
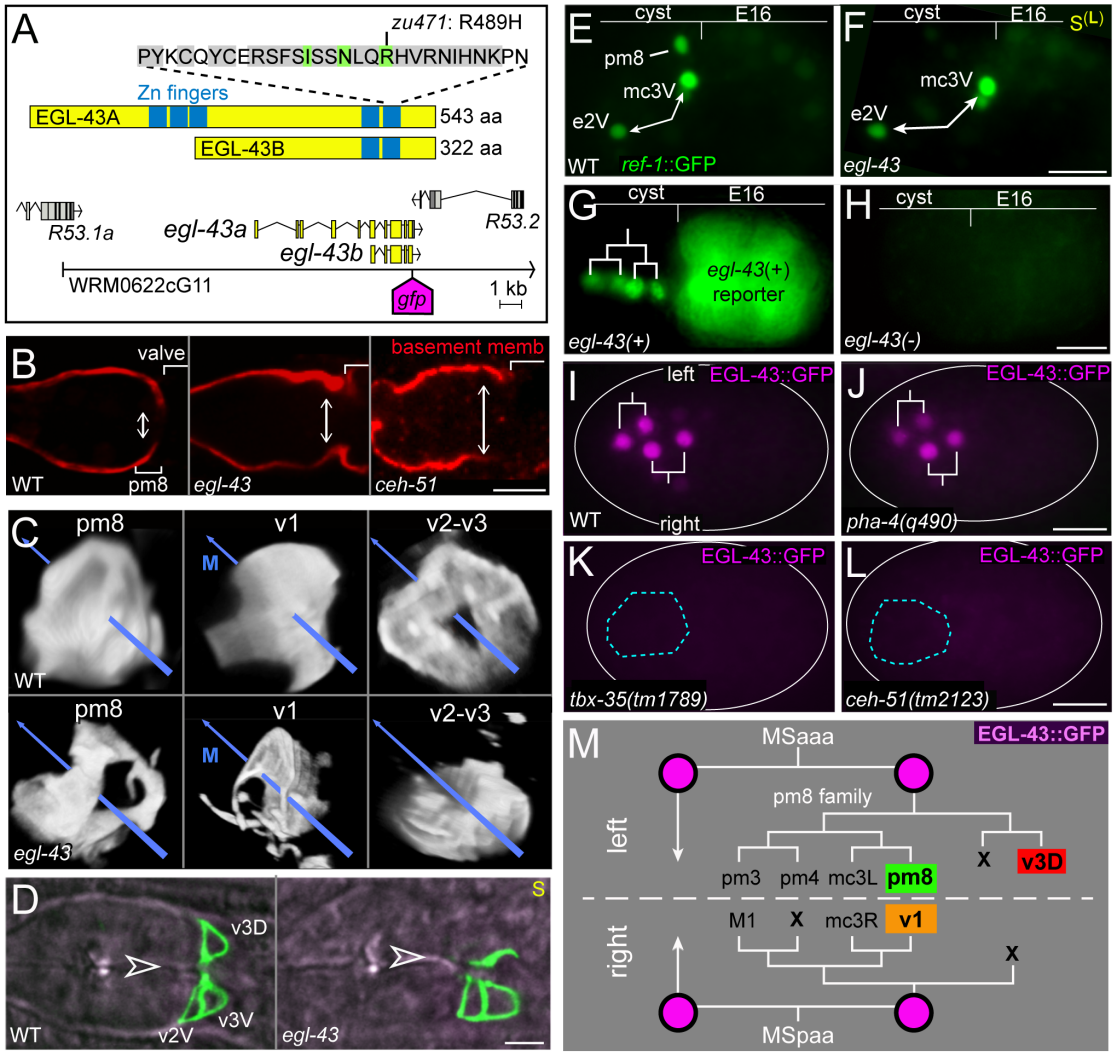
EGL-43 expression and *egl-43* mutant phenotypes. (A) Diagram of the EGL-43 protein and *egl-43* gene. WRM0622cG11 is a fosmid that rescues the *egl-43(zu471)* mutant; the site of *gfp* insertion for the EGL-43::GFP fosmid reporter is indicated. (B) Wild-type, *egl-43(zu471)* and *ceh-51(tm2123)* larvae immunostained for the basement membrane component UNC-52/Perlecan (red). The wild-type, but not mutant, basement membrane curves deeply inward, following the posterior surface of the pm8 cell body and nearly separating the pharynx from the valve (see [22]). (C) Wild-type and *egl-43* larvae expressing reporters as listed for either pm8 (*ref-1* ^153 bp^::GFP::CAAX), v1 (*egl-43*::GFP::CAAX), or v2-v3 (*mig-13*::MIG-13::GFP). Images are surface renderings as for Video S3, rotated to show posterior views of each cell(s); the midline is indicated by a blue arrow. Note the lack of v2-v3 cells dorsal to the midline in the *egl-43* mutant. (D) Images of hatched larvae expressing *mig-13*::MIG-13::GFP (green) and showing the apparent absence of the v3D valve cell in the *egl-43* mutant; the midline is indicated by an open arrowhead. (E,F) Live embryos showing EGL-43-dependent expression of *ref-1* ^1.8 kb^::REF-1::GFP in pm8. Notch-independent expression occurs in other pharyngeal cells, such as the sister cells e2V and mc3V (double-headed arrows); see [36]. (G,H) Live embryos showing the *egl-43(+)* dependence of a *lin-12*-derived reporter for the pm8 family (green nuclei, *lin-12* ^pm8^::HIS-GFP). The embryos are progeny of an *egl-43(zu471)/mIn1[dpy-10(e128) mIs14]* adult; the *mIn1* balancer contains *egl-43(+)* and a transgene that drives GFP expression in intestinal cells. Thus, only the embryo in G is *egl-43(+)*, and only this embryo expresses the *lin-12*-derived reporter (see Table 1 for quantification). (I,J) EGL-43 expression in a wild-type embryo (I) and a *pha-4(q490)* mutant embryo (J, n = 14/14 embryos with similar expression). (K,L) EGL-43::GFP is not expressed in the pharyngeal precursors (blue outline) of most *tbx-35* (K) and *ceh-51* (L) mutant embryos. In these experiments, the EGL-43::GFP transgene was marked with *end-1*::mCherry (not shown), and only mCherry-positive embryos were scored. (M) Partial lineage tree showing the MS descendants that express EGL-43::GFP, and showing the fates of these descendants in normal development (see [9]). Bars: (B–D) 5 microns; (E–L) 10 microns.

In parallel with the above studies on Notch targets, we used a genetic screen to isolate mutants with posterior mispositioning of pharyngeal nuclei, similar to the phenotypes of *lag-1; eff-1* double mutants and *ina-1*/alpha-integrin mutants (Figure 9A). Although most mutants examined had severe and general defects in development, two recessive, embryonic and larval lethal mutants, *zu470* and *zu471*, had relatively normal morphogenesis of non-pharyngeal tissues, and these were selected for molecular analysis. DNA sequencing showed that *zu470* contains a nonsense mutation in *ina-1*/alpha-integrin, and was not analyzed further in this study. Mapping and complementation showed that *zu471* is a new allele of *egl-43* (see Materials and Methods). EGL-43 is a zinc-finger transcription factor orthologous to the mammalian proto-oncogene EVI1, and *egl-43* mutants have defects in motor neuron migration and vulval morphogenesis [42]–[44]. DNA sequencing showed that *zu471* causes a R489H substitution in the C-terminal zinc finger domain; R489 is conserved in EVI1 proteins from nematodes to mammals, is predicted to contact DNA, and is essential for mammalian EVI1 to bind DNA in vitro (Figure 10A) [42], [45]. Embryos and larvae homozygous for *mnDf24*, a deficiency that removes *egl-43* [46], [47], closely resemble *egl-43(zu471)* homozygotes, suggesting that *zu471* is a strong loss-of-function allele (Table 1).

**Table 1:** *egl-43* regulates pm8 gene expression.

cells scored [reporter]	genotype	expressing animals (%)	# cells (range)	n
total pharyngeal and valve cells^†^ [PHA-4::GFP]	WT	100	86.3 (84–89)	6
	*egl-43(zu471)*	100	86.3 (85–88)	6
mc2 and mc3 [*pax-1*::GFP]	WT	100	6	46
	*egl-43(zu471)*	100	5.5 (4–7)	35
pm5–pm8^†^ [*myo-2*::GFP]	WT	100	13	50
	*egl-43(zu471)*	100	13.5 (11–16)	15
v1 [EFF-1::GFP]	WT	100		50
	*egl-43(zu471)*	100		10
pm8 [*aff-1*::GFP]	WT	98		53
	*egl-43(zu471)*	44		57
pm8 [*ref-1* ^1.8 kb^::GFP]	WT	100		48
	*egl-43(zu471)*	48		38
pm8 [*pax-1*::GFP]	WT	100		46
	*egl-43(zu471)*	17		35
pm8^†^ [*ceh-24*::GFP]	WT	100		80
	*egl-43(zu471)*	52		222
	*mnDf24*	53		246
pm8 lineage [*lin-12* ^pm8^::GFP]	WT	99		345
	*egl-43(zu471)*	0		63
	*pha-4(q490)*	0		44
	*ceh-51(tm2123)*	0		11

Reporter gene expression was scored in embryos between 300–450 minutes or in L1 animals (indicated by ^†^).

Most *egl-43(zu471)* mutants appeared to have the correct numbers of several specific cells including marginal cells (mc2, mc3), muscles (pm5–pm8), and some valve cells (v1) (Table 1). As predicted from the posterior mispositioning of pharyngeal nuclei, we found that the pharynx-valve boundary did not form properly in *egl-43(zu471)* animals, with the pharyngeal basement membrane failing to extend between pm8 and v1 (double-headed arrows in Figure 10B, compare with Figure 5K). pm8 and v1 had relatively normal positions in the mutant larvae, and appeared to surround the midline. However, both cells usually appeared perforated, and had variable and abnormal processes extending anterior and/or posterior from the cell body (15/16 larvae, Figure 10C). *egl-43(zu471)* larvae expressing a reporter for both the v2 and v3 valve cells showed cell number and/or positioning defects for these cells (25/25 animals), with the defect often appearing to result from the mispositioning or absence specifically of the dorsal valve cell, v3D (11/25 animals; Figure 10C, 10D). In *egl-43(zu471)* embryos, pm8 and the valve cells appeared to be in their normal positions at the double plate stage. However, v3D did not spread dorsally at the left-right boundary of intestinal cells, and instead remained in the interior of the cyst (n = 2 embryos; Figure 3H) similar to *die-1* mutant embryos (Figure 3G). At later stages, when v2L and v2R normally spread dorsally along the int1 surfaces before intercalating anterior to v3D (Figures 6G and S3C, S3D), the v2L and v2R cells in *egl-43* mutant embryos continued dorsally along the intestinal cells (Figure S3F).

In examining *egl-43* mutants with additional reporters for pm8, we noticed that pm8 lacked or showed variable expression of several genes (*ref-1*, *aff-1*, *pax-1*, *ceh-24*; Figures 9C and 10F, Table 1). All of these genes are LIN-12/Notch targets. For example, *ref-1* is expressed in the cyst cells e2V, mc3V, and pm8 (Figure 10E), but only pm8 expression is dependent on LIN-12/Notch [22]. In *egl-43* mutants, *ref-1* was expressed in e2V and mc3V, but not in pm8 (Figure 10F). We found that reporters driven by the *lin-12* ^pm8^ enhancer were not expressed in *egl-43* mutant embryos, suggesting a defect in LIN-12/Notch expression (Figure 10G, 10H, Table 1). The *lin-12* ^pm8^ enhancer contains a predicted EVI1 binding site (TCCGGT) [48], [49] that is conserved in several related *Caenorhabditis* species, however, mutation of this site to CGCTGT did not abrogate reporter expression (data not shown). We next scored LIN-12 expression directly by immunostaining the progeny of heterozygous *egl-43* parents, using a balancer marked with a fluorescent reporter (see Materials and Methods). Only 8.8% of embryos with LIN-12 expression in the pm8 family were homozygous for the *egl-43* mutation, indicating that EGL-43 contributes to LIN-12 expression in these cells (n = 23/262; *p*<0.01, chi-squared test).

We constructed a rescuing *egl-43*::EGL-43::GFP fosmid reporter (Figure 10A) to determine the expression pattern of EGL-43. In addition to neuronal expression described previously [47], EGL-43::GFP was expressed at about 150 minutes in four descendants of a blastomere called MS; these descendants are pharyngeal/valve precursors (Figure 10I, 10M). Surprisingly, PHA-4/FoxA was not required for EGL-43::GFP expression in these precursors (Figure 10J), in contrast to most other examples of pharyngeal gene expression [40], [50]. EGL-43::GFP was not detectable in most embryos lacking either of the transcription factors TBX-35 (22/36 negative, Figure 10K) or CEH-51 (32/34 negative, Figure 10L), which have partially redundant functions in regulating PHA-4/FoxA [51], [52]. We found that *ceh-51(tm2123)* mutants had apical junction and basement membrane defects in the valve that closely resembled *egl-43* mutants, while *tbx-35(tm1789)* mutants had junctional defects throughout the pharynx (Figure 10B and data not shown).

In wild-type development, the anterior two MS descendants that express EGL-43::GFP do not contribute cells to the posterior pharynx or valve. However, the posterior MS descendants produce pm8, v1, and v3D, each of which are defective in *egl-43* mutants (Figure 10M) [9]. These posterior blastomeres also produce the dorsal mc3 marginal cells (mc3L and mc3R), but not the ventral mc3 cell or any of the mc1 or mc2 marginal cells. We noticed that the dorsal mc3 nuclei in *egl-43* mutant embryos typically had less expression of *pax-1*::GFP than wild-type mc3 nuclei, while the mc1 and mc2 nuclei appeared to have normal levels. For example, a wild-type, dorsal mc3 nucleus has a level of *pax-1*::GFP that is often higher (47%) and never less than the adjacent mc2 nucleus (upper #3 and #2 nuclei in Figures 9B and 7F; n = 48 embryos). By contrast, the dorsal mc3 nuclei in *egl-43* mutants often expressed less (43%) and never more than mc2 nuclei (upper #3 and #2 nuclei in Figure 9C; n = 38 embryos). Thus, *egl-43* mutants have pharynx-valve defects that stem from defects in LIN-12/Notch expression in MS descendants, and additional defects in closely related cells such as v3D, mc3L and mc3R that do not undergo Notch signaling.

## Discussion

*C. elegans* embryogenesis assembles a complex animal from a remarkably small number of cells. The six valve cells have the tasks of both forming a tube and linking two separate tubes, the pharynx and intestine. The pharynx and intestine develop from separate primordia that (1) form lumenal axes independently and at different times in development, and (2) change shape markedly during morphogenesis. The tubular shape of the pharynx results from apical constrictions that reshape cuboidal cells into wedge-shaped cells, collectively transforming the double plate primordium into a cyst. We showed that the valve originates from a disc of wedge-shaped cells at the posterior of the cyst; these cells continue to reposition and change shape, reducing the cross sectional area of the posterior disc to three or fewer cells, and reshaping the posterior disc into a short, thin tube (Figure 1). The examples of repositioning we observed did not lead to the migration of entire cells, but rather to the spread or shifting of bulk cytoplasm relative to the circumference of the cyst. Indeed, each cell in the pm8 family, and many if not all valve cells, appears to maintain a radial axis of apicobasal polarity while repositioning. Repositioning events begin with lateral lamellipodia that intercalate between neighboring cells, followed by the nucleus and/or cytoplasm. These circumferential intercalations are topologically similar to intercalations that occur in epithelial sheets. For example, hypodermal cells in *C. elegans* intercalate using subapical, basolateral protrusions after first developing apicobasal polarity [53]. The intercalation of polarized cells has also been observed in other examples of tubulogenesis, including zebrafish blood vessels [5] and Drosophil*a* tracheal tubes [6]. For tracheal tube development, cell intercalation requires components of the apical extracellular matrix [6]. Although pharyngeal and valve cell intercalations in *C. elegans* occur when their apical surfaces are very small (Figure 7E), laminin can be detected at the midline of the polarized cyst and intestine after these primordia polarize (Figure 5E), and by electron microscopy extracellular materials are visible adjacent to the apical surfaces of cyst cells (our unpublished results). Thus, it is possible that these extracellular materials have some role in pharyngeal/valve morphogenesis.

We observed dynamic lamellipodia in cyst cells that differentiate into valve cells, three types of muscles (pm3, pm4, and pm8), or one type of marginal cell (mc3), suggesting that many if not most cyst cells develop lamellipodia after becoming polarized. Cells likely use these protrusions to scan their neighborhood for signals that influence subsequent morphogenesis, much as the filopodia or lamellipodia of migrating cells probe their environment for directional cues (reviewed in [54]). For example, the pm4 lamellipodium might encounter signals that direct the circumferential repositioning of pm4 into the lateral muscle group. Signaling molecules such as ephrins are broadly expressed in the pharyngeal cyst and could contribute to repositioning [55]. Previous studies in *C. elegans* showed that diverse embryonic cells whose fates were transformed by genetic or physical manipulation could migrate to join cells of similar fate elsewhere in the embryo, a phenomenon termed “cell focusing” [56], [57]. The signaling pathways responsible for cell focusing are not known, and those studies observed only anterior-posterior repositioning of transformed cells. However, the MS descendants that comprise nearly all of the cyst cells described here were not analyzed. Filopodia can mediate Notch signaling in Drosophila [58], and we found that some of the early lamellipodia from pm8 extend repeatedly onto a ligand-expressing cell. However, we do not know if those lamellipodia contribute to Notch signaling in pm8.

We found that the transcription factor DIE-1 is required for proper morphogenesis of the valve. Previous studies showed that *die-1* mutants have diverse defects, including defects in the intercalations of dorsal hypodermal cells, apical junctions, and left-right asymmetries in size and gene expression of two chemosensory neurons [28], [59]. We observed defects in multiple examples of cell intercalations in *die-1* mutants, including defects in intestinal cell intercalations that might secondarily cause defects in valve cell polarity and apical junction formation. These results suggest that DIE-1 has a broad role in the intercalation of many types of embryonic cells. Cells in *die-1* mutants develop transient protrusions, but show incomplete or no intercalation of bulk cytoplasm between neighboring cells. Cell intercalation is a complex event where existing adhesive partners must be exchanged for new ones, with forces generated to displace cell mass (reviewed in [60]). It is possible that DIE-1 has a general role in regulating cell adhesion or in the generation of adhesion-dependent force. For example, the basal surfaces of differentiated intestinal cells in *die-1* embryos appear abnormally rounded instead of flattening against the surrounding basement membrane (our unpublished observations). Transcriptional targets of DIE-1 that function in cell intercalation have not been identified, but DIE-1 both regulates, and is regulated by, microRNAs during neuronal development [28], [59].

### The Role of v3D in Aligning the Valve with the Intestine

Our results suggest that the valve cell v3D has an early and special role in forming the valve and aligning the valve with the intestine. We showed that v3D undergoes unique changes in cell shape at the double plate stage, when neighboring pharyngeal and valve cells remain cuboidal. The v3D cell body becomes embedded in the left-right boundary of the int1^p^ cells, whose later dorsal-ventral division produces the int1 ring of intestinal cells. Thus, v3D is pre-positioned at one of the two intestinal cell boundaries that define the crosshair positioning of the intestinal lumen. We showed that proper v3D development requires the transcription factors DIE-1 and EGL-43; in mutants defective in either factor, v3D does not extend along the left-right boundary of the intestinal cells. After the wild-type v3D docks at the left-right boundary, pm8 and v1 move into bilaterally symmetrical positions, flanking v3D. Symmetry with respect to the intestinal boundary per se does not appear essential for pm8 and v1 morphogenesis, as both cells appear to form normally in embryos lacking the intestine. However, symmetry might facilitate intercalation of the ventral valve cells that move dorsally along the surfaces of pm8 and v1, and that must do so in the short period before the embryo elongates and begins muscle contraction. For example, we showed that mutants lacking HMR-1/E-cadherin have defects in the dorsal intercalation of the v2L and v2R cells, and these mutants subsequently develop gaps or breaks in apical junctions within the valve.

### The Role of the Intestinal Primordium in Valve Formation

Cells throughout the digestive tract must have a common, radial axis of apicobasal polarity to form a continuous lumen; laminin is required to coordinate polarization of the pharyngeal primordium, but not the intestinal primordium [12]. Laminin localizes to the perimeter of the double plate primordium, except at the posterior surface which contacts the intestinal primordium. We showed here that intestinal cells prevent the posterior accumulation of laminin. The intestinal cells might simply block laminin access by adhering to the pharyngeal cells, or influence laminin accumulation through a more specific signaling pathway. Previous studies of laminin mutants showed that expression of laminin only in intestinal cells was sufficient to rescue their defects in apicobasal polarity, but it is not known which intestinal surfaces secrete laminin in those experiments [12]. In *die-1* mutants, laminin accumulates inappropriately on the posterior surfaces of ventral double plate cells, including the v2 valve cells (v2L and v2R). We propose that this defect results from the failure of the int2 intestinal cells to intercalate ventrally, between the parents of the v2 cells and the germ cell precursors. At later stages, some of the pharyngeal or valve cells in *die-1* mutants develop an axis of apicobasal polarity that is oblique to the radial axes of other pharyngeal or intestinal cells. Together, these results suggest that ectopic, posterior laminin has the potential to cue the inappropriate, anterior localization of apical proteins such as PAR-6. By preventing laminin accumulation, the intestinal cells ensure that posterior valve cells have the same, radial axis of apicobasal polarity as other cells in the digestive tract.

### Patterning of Cell Intercalations

As lamellipodia track circumferentially through the cyst they appear to follow invariant trajectories, intercalating between certain pairs of cells while bypassing others. For example, pm8 and v1 occupy bilaterally symmetrical positions in the cyst and extend symmetrical dorsal, and then ventral, lamellipodia. The ventral lamellipodia intercalate between bilaterally symmetrical pairs of cells (pm7/int1 then pm7/v2) before reaching the ventral marginal cell, mc3V. At or near mc3V, pm8 always crosses anterior to v1. The posterior face of mc3V is associated with a similarly shaped tract of laminin that forms shortly before, and disappears after, pm8 wrapping, and both laminin and a candidate laminin receptor, INA-1/integrin, are essential to form the pharynx-valve boundary (this report and [22]). Moreover, HMR-1/E-cadherin appears enriched at the boundary between pm8 and mc3V, apparently coincident with the laminin tract. Thus, adhesion to either the tract or mc3V might allow the ventral lamellipodium from pm8 to cross asymmetrically, anterior to the lamellipodium from v1.

Shortly before forming their ventral lamellipodia, pm8 and v1 extend dorsal lamellipodia that meet and stop, without crossing, on the anterior face of v3D. The zone of contact with v3D is highly enriched for HMR-1/E-cadherin, although HMR-1 is localized primarily to the apical surfaces of other cyst cells at this stage. HMR-1 enrichment between v3D and pm8 or v1 does not mediate permanent adhesion, as v2L and v2R subsequently intercalate between v3D and pm8 or v1. Instead, we speculate that adhesion to v3D might prevent the dorsal lamellipodia of pm8 and v1 from crossing randomly before the proper intercalation order is established by events on the ventral side of the cyst. In addition or alternatively, HMR-1/E-cadherin might participate in adhesive complexes that allow the subsequent intercalation of the v2L and v2R cell bodies. We showed that v2L and v2R can extend thin lamellipodia between v3D and either v1 or pm8 in mutants lacking embryonic expression of HMR-1/E-cadherin, but v2L and v2R fail to shift their nuclei and bulk cytoplasm. We do not yet know whether mutants lacking both embryonic and maternally supplied HMR-1 have more severe valve defects; such embryos have general defects in body morphogenesis that result in the rupture and disintegration of internal tissues, and were not analyzed in this study [33].

### Notch Signaling and EGL-43/EVI1 in Pharynx-Valve Development

We propose that the asymmetry of Notch signaling dictates that the ventral lamellipodium from pm8 must cross anterior to v1. pm8 and v1 are lineal homologs, which are non-sister cells born from similar sublineages, and many such cells in *C. elegans* have equivalent development potential. However, only pm8 expresses the LIN-12/Notch receptor, and only pm8 expresses Notch target genes. We found no evidence that Notch signaling regulates the basic morphogenetic events of pm8 intercalation and wrapping, which mirror the Notch-independent morphogenesis of v1. Instead, Notch signaling likely regulates multiple pharyngeal-specific features of pm8. Previous results showed that pm8 fails to express a pharyngeal myosin-specific reporter in Notch mutants [22], and pharyngeal muscle expression has been described for several genes that we identified here as candidate Notch targets. Moreover, we showed that mutant larvae defective in *inx-20*, a validated Notch target in pm8, fail to coordinate pm8 muscle contraction with that of other muscles. Since only pm8 has the potential to become a muscle, it must cross anterior to v1 in order to join other pharyngeal muscles.

Notch signaling generates a nuclear localized, ternary complex of proteins that includes CSL proteins such as LAG-1 in *C. elegans* (reviewed in [61]). Our results suggest that several genes with predicted, conserved binding sites for both LAG-1/CSL and PHA-4/FoxA binding sites are likely to be direct Notch targets in pm8. The subset of genes that contained both predicted sites but that did not show expression in pm8 might instead be targets in other Notch interactions. For example, PHA-4/FoxA is expressed in the intestine and rectal epithelium during some stages of development, and both of these tissues undergo Notch signaling [15], [62]. Transcription factors other than PHA-4/FoxA might also collaborate with LAG-1 in driving Notch expression in pm8. Previous studies identified an enhancer in the *ceh-24* gene that can drive Notch-dependent expression in pm8 [22], [63]; this enhancer lacks an obvious binding site for PHA-4/FoxA, but mutational analysis identified other conserved sites that are critical for pm8 expression [63]. Moreover, EGL-43 provides an example of a transcription factor that is expressed in the pm8 lineage independent of PHA-4. Thus, different target genes could be regulated combinatorially in pm8 by LAG-1 plus PHA-4/FoxA, or by LAG-1 plus at least one other transcription factor. Notch is activated in pm8 shortly before morphogenesis, and studies in both *C. elegans* and Drosophila indicate that direct Notch targets can be expressed within minutes after signaling [35], [64]. However, several direct Notch targets are first detectable in pm8 as late as five hours after signaling, and similar, late expression of some Notch targets has been described recently in Drosophila [64]. We showed that the LAG-1 protein remains in the nucleus of pm8 long after signaling, raising the possibility that LAG-1 might remain at target gene promoters for long periods of time. Previous studies noted a high density of LAG-1 binding sites within the *lag-1* gene itself [65], and we speculate that these sites might contribute to persistent LAG-1 expression

We showed that LIN-12/Notch expression depends, at least in part, on the transcription factor EGL-43. EGL-43::GFP expression in pharyngeal and valve precursors does not require PHA-4/FoxA, but requires two other transcription factors, TBX-35 and CEH-51. These data indicate that a PHA-4-independent pathway influences pharyngeal cell differentiation through EGL-43, and one likely candidate is the POP-1/TCF pathway [66]. Sister cells born in anterior-posterior divisions throughout the embryo have unequal levels of nuclear POP-1/TCF, including the pharyngeal cells that express EGL-43 (Figure 10M), their daughters, and at least some later descendants of these cells [66], [67]. POP-1/TCF is thought to collaborate with a variety of transcription factors in regulating cell fate, and POP-1 function in the pharynx has been shown to regulate pharyngeal muscle specification [68]. We showed that neighboring cells can undergo complex and very different programs of morphogenesis during the development of the valve tube, and this complexity could depend on systems like POP-1 that can generate transcriptional diversity between closely related, or even sister, cells.

## Materials and Methods

### Strains, Transgenes, Isolation and Mapping of *zu471*

See Text S1.

### Imaging and 3D Modeling

Embryos were mounted as described [9]. Time-lapse movies were acquired with a Hamamatsu C9100 camera on a Nikon TE-2000 inverted microscope equipped with a Yokogawa CSU-10 spinning disk and running Volocity 5.3.3 (Improvision). The absolute times of the image sequences were scaled relative to either the division of int1^p^ to int1 at 350 minutes or the pm8 nucleus reaching the ventral perimeter of cyst at 430 minutes [9]. For 3D modeling of the double plate to cyst transition, movies were taken of developing embryos and cell contours were generated from Z stacks of each time point using TrakEM2 software [69]. The mesh model was reduced using quadratic edge collapse decimation (MeshLab open source software; http://meshlab.sourceforge.net/), and viewed with Photoshop 3D animation (Photoshop CS5). Surface images of cell shapes as in Video S2 were rendered from Z stacks using Volocity 3D Opacity software.

### Immunostaining

The following antibodies/antisera were used: anti-LAG-1 (gift from Judith Kimble), anti-LIN-12 (gift from Stuart Kim), anti-UNC-52 [70] (gift from Don Moerman), MH27 [71], anti-laminin (mAbGJ2) [22] and anti-GFP (ab6556, Abcam). Worm and embryo fixation procedures were performed essentially as described [11].

### Bioinformatics

We used a set of custom perl scripts to identify conserved predicted LAG-1/CSL sequences in WormBase release WS230 of the *C. elegans*, *C. brenneri*, *C. briggsae*, *C. japonica*, and *C. remanei* genomes [38], [72]. We limited our analysis to the 15,752 *C. elegans* genes with single orthologs in at least two other *Caenorhabditis* genomes. We searched 5 kb upstream of these genes and their orthologs for occurrences of the sequence RTGGGAA, which has previously been shown to be bound by LAG-1 [65]. We conducted pairwise alignments of these upstream regions using PhyME [73] to identify conserved sequence blocks (regions of at least 14 nt with at least 70% identity). We considered a predicted LAG-1/CSL sequence conserved if it aligned to blocks containing RTGGGAA sequences from at least two other *Caenorhabditis* genomes. Multiple sequence alignments were performed using MLAGAN [74] and edited with Jalview (http://www.jalview.org).

### Cell Ablations

A 440-nm laser microbeam (Photonics Instruments) was used to ablate the E blastomere. Immediately following ablation, embryos were removed from the microscope slide and allowed to develop without compression on an agar filled petri dish.

## Supporting Information

Figure S1 Formation and remodeling of the cyst. (A) Diagram of the pharynx, the valve, and the anterior intestine will full cell names [9]. Optical planes used throughout this study are Horizontal (H) = Anterior-Posterior×Left-Right, Sagittal (S) = Anterior-Posterior×Dorsal-Ventral, and Transverse (T) = Dorsal-Ventral×Left-Right. (B) Images of a live embryo labeled as in Figure 2B, but taken at a horizontal optical plane through the roof of the double plate (left) and later cyst (right). Partial cell identity diagrams for the double plate and cyst are shown below each image. The apical constriction of double plate cells, and a general dorsal shift in cell positions, flattens the roof and brings several marginal cells into the optical plane. While many cells retain their neighbors between the double plate and cyst stages, several small but reproducible shifts in cell positions are evident. For example, pm7D intercalates between the neurons I6 and M5, then intercalates posterior to pm6D. M5 spreads to form a distinctive, terminal “cap” at the dorsal midline. The pm8 family is visible from the roof, except for pm4L, which has shifted laterally (dashed white line). (C) Horizontal, dorsal plane as in panel B of an embryo expressing membrane and nuclear reporters for the pm8 family (green, *lin-12* ^pm8^::mCherry::CAAX; red, *lin-12* ^pm8^::HIS-GFP) and a general pharyngeal membrane reporter (red, *mig-13*::MIG-13::GFP). A lamellipodium from pm8 extends to the right, intercalating between pm7D and v3D, and then between M5 and v3D, until it reaches a mirror image lamellipodium that extends from v1. Bars: (B) 10 microns, (C) 2.5 microns.
(TIF)

Figure S2 Dynamics of PAR-6 and HMR-1 localization near the midline. (A, B) Image sequences showing changes in HMR-1::GFP (green) and PAR-6::mCherry (red) localization as the double plate transforms into the cyst, taken at a horizontal plane through the midline; images in panel B are of the bracketed region indicated in panel A, and the arrowhead indicates the junction of the double plate or cyst with the intestinal primordium. To show the entire midline, each image in panel B is a maximum intensity projection through 1.5 microns. Note that the initial PAR-6 expression in the pharyngeal primordium is not coaxial with PAR-6 expression in the intestinal primordium (arrows). Shortly before the int1^p^ cells divide at 350 minutes, PAR-6 disappears from the midline-facing surfaces of these cells (bracket at 350 minutes), then later reappears as the int1 ring forms and links with the valve cells (not shown). (C) Image sequence from the same embryo shown in panel A showing a horizontal plane through the midline (M) at the interface between the pharynx and the intestine. The v3D cell body appears to shrink over time in this focal plane because the bulk cytoplasm moves to a higher (dorsal) level (compare with sagittal view in Figure 3F). Note that the left and right int1^p^ cells have concentrated PAR-6 and HMR-1 on the membranes adjacent to v3D (closed arrowheads) compared to contacts with other double plate cells (open arrowheads). HMR-1 remains enriched around the body of v3D as it moves dorsally, eventually leaving only a small process in this optical plane (arrow). Bars: (A–C) 6 microns.
(TIF)

Figure S3 Circumferential intercalations of pm8, v1, v2L and v2R. Image sequences show cell intercalations as indicated; cell identification drawings are shown beneath each sequence, and the color key is shown in panel A. In addition to other reporters listed below, the embryos express a reporter for pharynx/valve membranes (panels B, C; red, *mig-13*::MIG-13::GFP) or for all membranes (panels D–F; red, *pie-1*::mCherry::PH(PLC1∂1)). (B) Embryo expressing a membrane reporter specific for the pm8 family (green, *lin-12* ^pm8^::mCherry::CAAX). Note that the lamellipodium from pm8 (arrow) can be tracked by either the specific reporter for the pm8 family (green), or by the non-specific membrane reporter (red), presumably because of the local increase in membrane density. Similar increases in membrane fluorescence are observed for other intercalations in the cyst (see arrows in panel C). (C) Right sagittal side of cyst showing intercalation of v1 and v2R; see also Video S4. Note that the bulk of the v2R cell body has intercalated between v1 and v3D by 410 minutes. (D) Embryo expressing reporters for marginal cell and pm8 nuclei (green nuclei, *pax-1*::HIS-GFP) and for intestinal nuclei/cytoplasm (green, F22B7.9::GFP). v2L (dark blue) first becomes visible in the optical plane at 400 minutes, as it spreads outward to the periphery of the cyst and begins migrating dorsally. Note that much of the v2L cell body has intercalated between pm8 and v3D by 424 minutes. (E) HMR-1::GFP localization during pm8 and v2L (asterisk) intercalations. This embryo also expresses the same marginal cell nuclear reporter (green nuclei) as in panel C (3 = mc3V and mc3L). In the diagram, HMR-1 is indicated in black, and cell colors are reduced in intensity after the first panel for better contrast with HMR-1. Note the lack of HMR-1::GFP around the v2V and v3V cells, which do not undergo nuclear migration. (F) Intercalation of v2R in an *egl-43* mutant. v2R (blue) remains at the interface between v1 and an int1 cell, and the v3D cell is not visible. Bars: (B–F) 2.5 microns.
(TIF)

Figure S4 Control of Notch-dependent gene expression in pm8. (A) Diagram of reporter constructs showing conserved LAG-1/CSL and FoxA sequences in orthologous sequences from related nematodes. To test for Notch dependence, pm8 expression was scored in either (^a^) *lin-12(n941) glp-1(q46)* or (^b^) *lag-1(q385)* embryos as indicated [n = 20–69 for WT; n = 14–98 for *notch(-)*]. (B–D) *pax-1*::HIS-GFP expression in the pharynx. (B) Expression of the wild-type *pax-1* transgene as in Figure 9B. (C) Expression after mutating the candidate LAG-1 binding site (GTGGGAA) to GAGGCAA; expression persists in the marginal cells, but is absent from pm8. The dorsal nucleus indicated by an asterisk is v1. (D) Differential interference contrast (upper) and fluorescence (lower) micrographs of an embryo expressing a *pax-1* transgene in which candidate FoxA sites 1 and 2 have been mutated to TATATGG and TATATGT, respectively. (E) EMSA using a previously described probe that contains two LAG-1 binding sites [43]. Competitor DNA contains either wild-type sequence from *inx-11* or a sequence in which the ATGGGAA site has been mutated to AAGGCAA. Bars: (B–D) 2.5 microns.
(TIF)

Table S1 Positions of the conserved LAG-1 sequences identified. Coordinates refer to the WS230 genome assembly. Two genes are listed in cases where the predicted LAG-1 site was identified in a region between genes oriented head-to-head. Genes annotated as having general pharyngeal or pharyngeal muscle expression are indicated (*).
(XLSX)

Text S1 Supplemental Materials and Methods.
(DOCX)

Video S1 Development of the pharynx/intestine interface. 3D models of cells at the interface between the pharyngeal and intestinal primordia.
(MOV)

Video S2 Dynamic lamellipodia in polarized cyst cells. Time-lapse movies beginning around 330 minutes of the pm8 family as in Figure 6B. The movie shows simultaneous views of two orthogonal planes centered on the mc3DL nucleus. Note that mc3DL is already wedge shaped (polarized) at the start of the sequence, and maintains this basic shape as the various lamellipodia extend from the lateral membranes (viewed in the horizontal plane). Asterisks indicate positions of cells (not shown) that express LAG-2, a ligand for LIN-12/Notch (see also [22]).
(MOV)

Video S3 pm8 family morphogenesis. 3D surface rendering of a time-lapse movie of the pm8 family; reporters as in Figure 7A.
(MOV)

Video S4 v2R intercalation. The movie shows a right sagittal optical plane of an embryo as in Figure 2B. The boxed interface region is shown in the lower panels. At 360 minutes the int1^p^ cells divide, and v2R intercalation is visible 24 minutes later.
(MOV)

Video S5 Pharyngeal pumping. A time-lapse differential interference contrast movie of pharyngeal pumping in wild-type and *inx-20(ok681)* L1 larvae. Note that the opening and closing of wild-type pm8 is coordinated, with a slight delay, to the opening and closing of pm7. In the *inx-20(ok681)* larva, the opening and closing of pm8 is not coordinated to the opening and closing of pm7, resulting in the regurgitation of bacteria from the intestine.
(MOV)
