# Supplementary figures and images for: Cell Interactions and Patterned Intercalations Shape and Link Epithelial Tubes in C. elegans
Source: PLoS Genet. 2013 Sep 5;9(9):e1003772. doi: 10.1371/journal.pgen.1003772 (PMC3764189; doi:10.1371/journal.pgen.1003772)

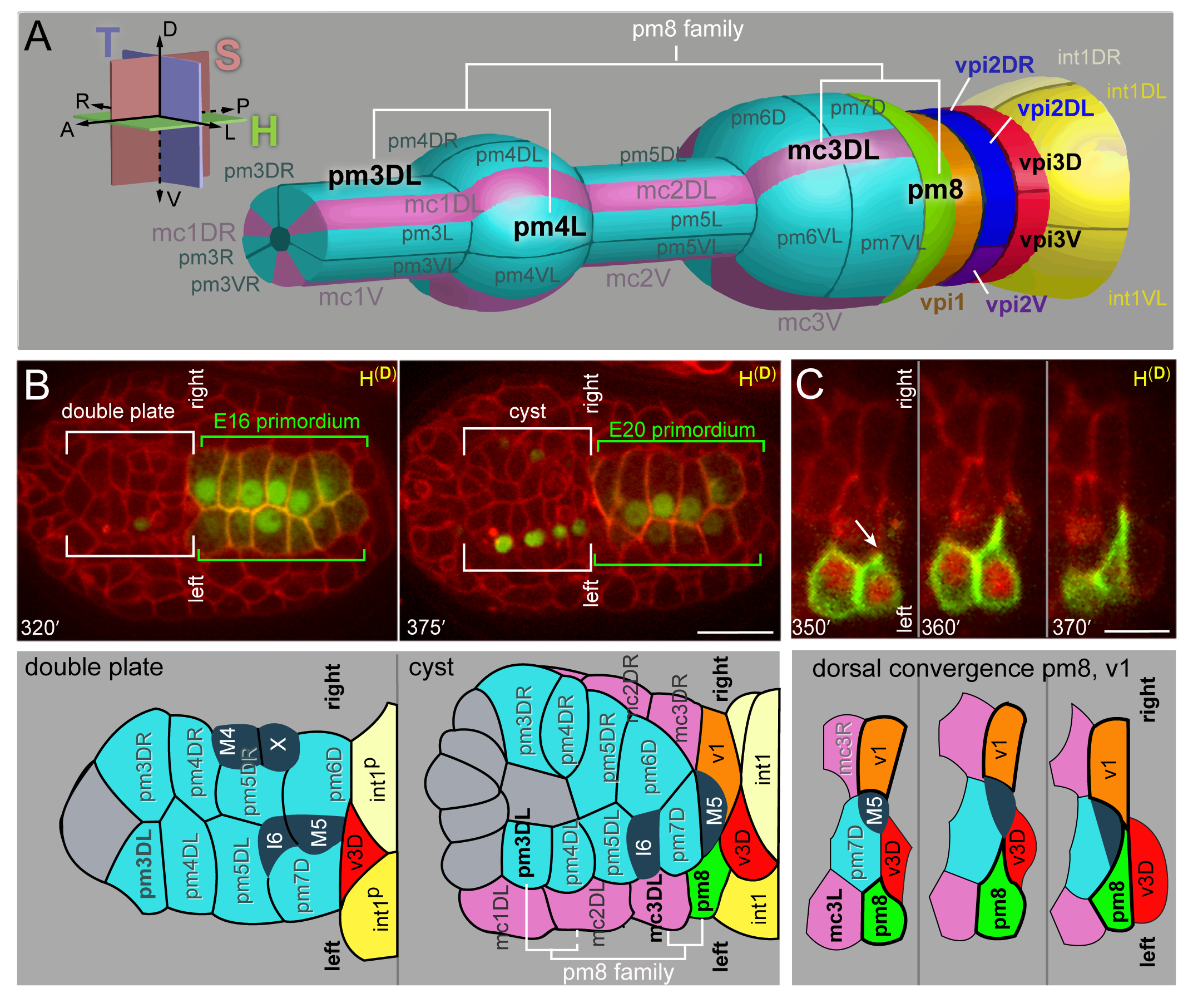

Supplement: Figure S1 — Formation and remodeling of the cyst. (A) Diagram of the pharynx, the valve, and the anterior intestine will full cell names [9]. Optical planes used throughout this study are Horizontal (H) = Anterior-Posterior×Left-Right, Sagittal (S) = Anterior-Posterior×Dorsal-Ventral, and Transverse (T) = Dorsal-Ventral×Left-Right. (B) Images of a live embryo labeled as in Figure 2B, but taken at a horizontal optical plane through the roof of the double plate (left) and later cyst (right). Partial cell identity diagrams for the double plate and cyst are shown below each image. The apical constriction of double plate cells, and a general dorsal shift in cell positions, flattens the roof and brings several marginal cells into the optical plane. While many cells retain their neighbors between the double plate and cyst stages, several small but reproducible shifts in cell positions are evident. For example, pm7D intercalates between the neurons I6 and M5, then intercalates posterior to pm6D. M5 spreads to form a distinctive, terminal “cap” at the dorsal midline. The pm8 family is visible from the roof, except for pm4L, which has shifted laterally (dashed white line). (C) Horizontal, dorsal plane as in panel B of an embryo expressing membrane and nuclear reporters for the pm8 family (green, lin-12 pm8::mCherry::CAAX; red, lin-12 pm8::HIS-GFP) and a general pharyngeal membrane reporter (red, mig-13::MIG-13::GFP). A lamellipodium from pm8 extends to the right, intercalating between pm7D and v3D, and then between M5 and v3D, until it reaches a mirror image lamellipodium that extends from v1. Bars: (B) 10 microns, (C) 2.5 microns. (TIF) [file pgen.1003772.s001.tif]

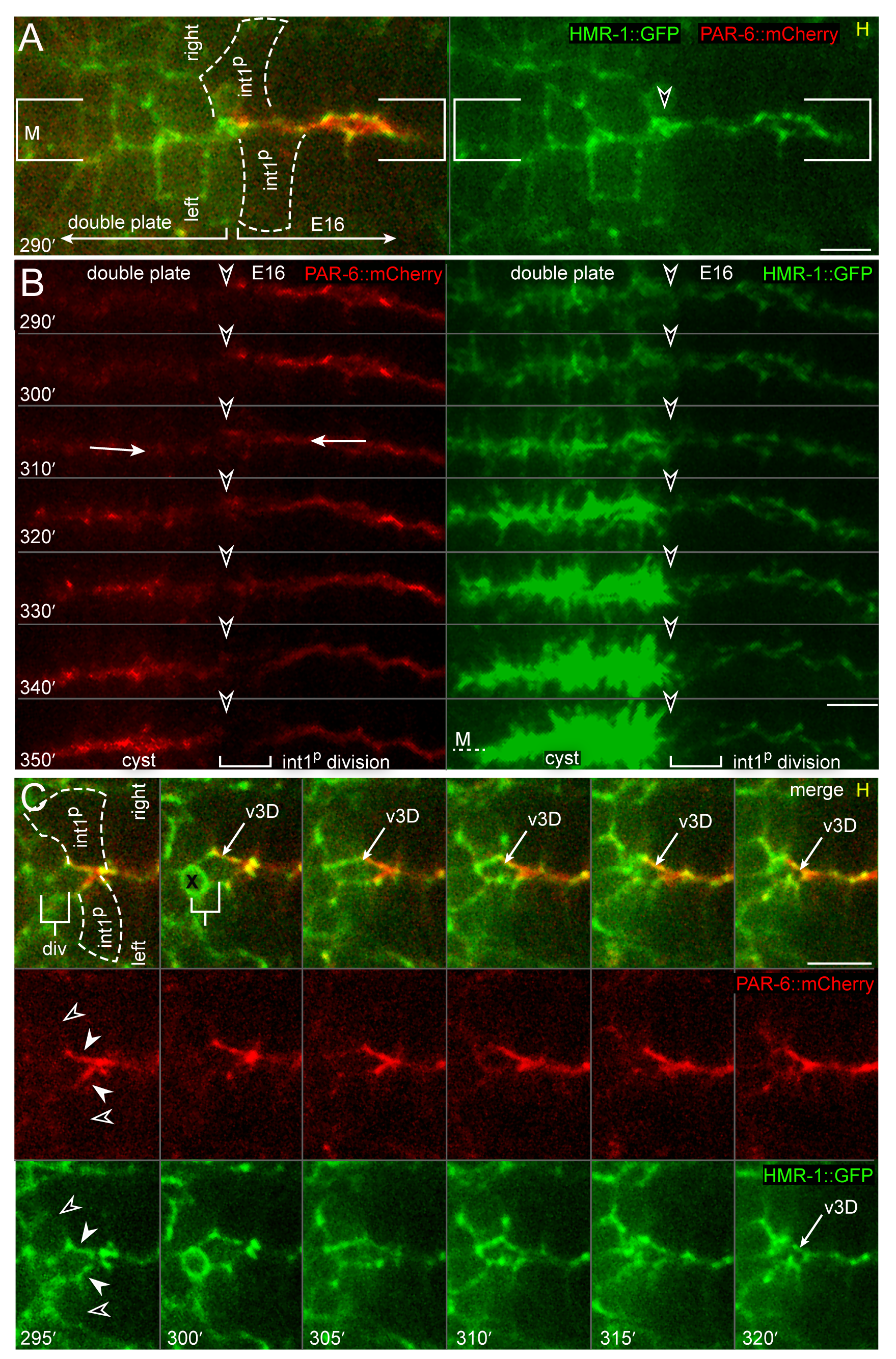

Supplement: Figure S2 — Dynamics of PAR-6 and HMR-1 localization near the midline. (A, B) Image sequences showing changes in HMR-1::GFP (green) and PAR-6::mCherry (red) localization as the double plate transforms into the cyst, taken at a horizontal plane through the midline; images in panel B are of the bracketed region indicated in panel A, and the arrowhead indicates the junction of the double plate or cyst with the intestinal primordium. To show the entire midline, each image in panel B is a maximum intensity projection through 1.5 microns. Note that the initial PAR-6 expression in the pharyngeal primordium is not coaxial with PAR-6 expression in the intestinal primordium (arrows). Shortly before the int1p cells divide at 350 minutes, PAR-6 disappears from the midline-facing surfaces of these cells (bracket at 350 minutes), then later reappears as the int1 ring forms and links with the valve cells (not shown). (C) Image sequence from the same embryo shown in panel A showing a horizontal plane through the midline (M) at the interface between the pharynx and the intestine. The v3D cell body appears to shrink over time in this focal plane because the bulk cytoplasm moves to a higher (dorsal) level (compare with sagittal view in Figure 3F). Note that the left and right int1p cells have concentrated PAR-6 and HMR-1 on the membranes adjacent to v3D (closed arrowheads) compared to contacts with other double plate cells (open arrowheads). HMR-1 remains enriched around the body of v3D as it moves dorsally, eventually leaving only a small process in this optical plane (arrow). Bars: (A–C) 6 microns. (TIF) [file pgen.1003772.s002.tif]

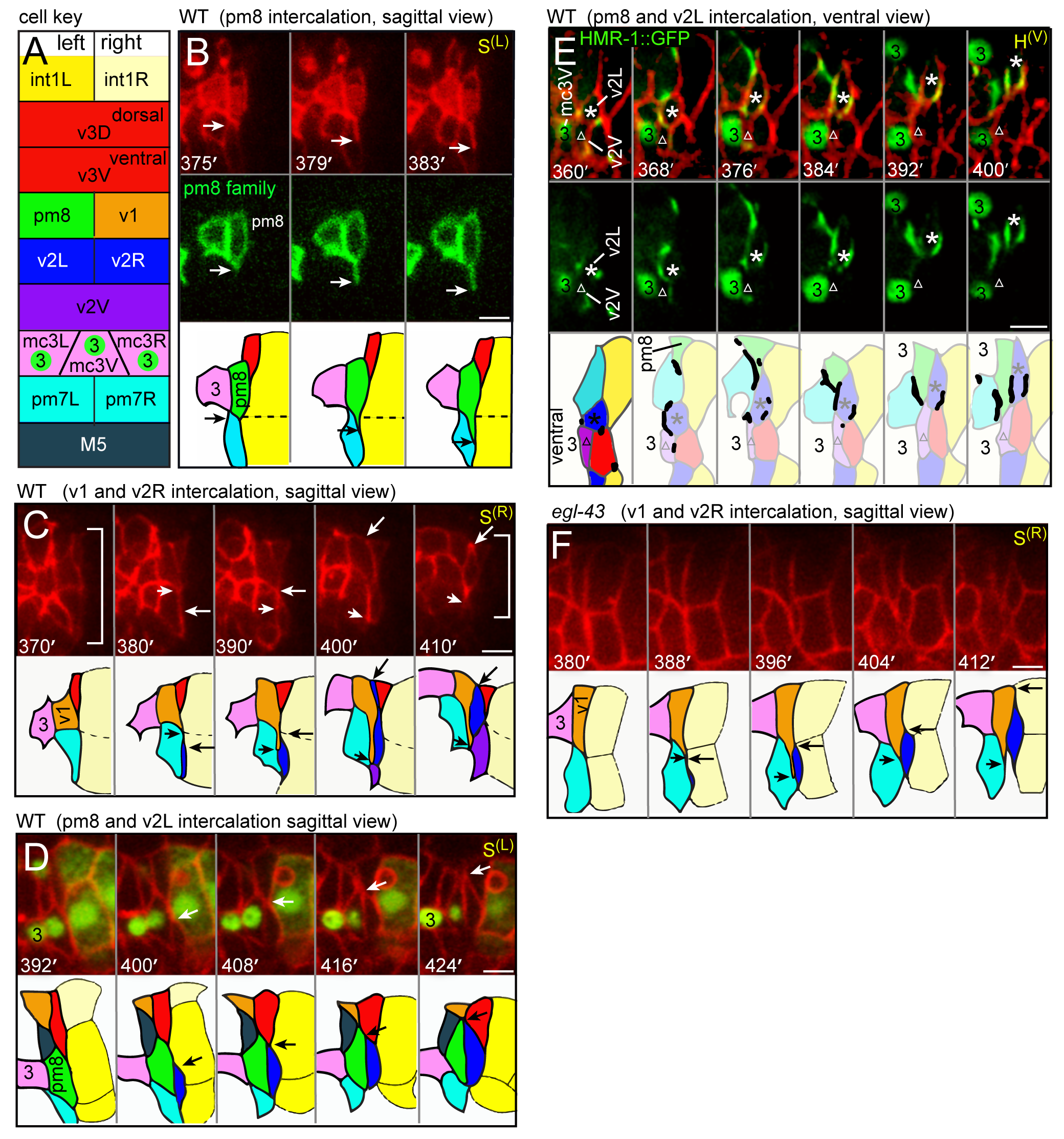

Supplement: Figure S3 — Circumferential intercalations of pm8, v1, v2L and v2R. Image sequences show cell intercalations as indicated; cell identification drawings are shown beneath each sequence, and the color key is shown in panel A. In addition to other reporters listed below, the embryos express a reporter for pharynx/valve membranes (panels B, C; red, mig-13::MIG-13::GFP) or for all membranes (panels D–F; red, pie-1::mCherry::PH(PLC1∂1)). (B) Embryo expressing a membrane reporter specific for the pm8 family (green, lin-12 pm8::mCherry::CAAX). Note that the lamellipodium from pm8 (arrow) can be tracked by either the specific reporter for the pm8 family (green), or by the non-specific membrane reporter (red), presumably because of the local increase in membrane density. Similar increases in membrane fluorescence are observed for other intercalations in the cyst (see arrows in panel C). (C) Right sagittal side of cyst showing intercalation of v1 and v2R; see also Video S4. Note that the bulk of the v2R cell body has intercalated between v1 and v3D by 410 minutes. (D) Embryo expressing reporters for marginal cell and pm8 nuclei (green nuclei, pax-1::HIS-GFP) and for intestinal nuclei/cytoplasm (green, F22B7.9::GFP). v2L (dark blue) first becomes visible in the optical plane at 400 minutes, as it spreads outward to the periphery of the cyst and begins migrating dorsally. Note that much of the v2L cell body has intercalated between pm8 and v3D by 424 minutes. (E) HMR-1::GFP localization during pm8 and v2L (asterisk) intercalations. This embryo also expresses the same marginal cell nuclear reporter (green nuclei) as in panel C (3 = mc3V and mc3L). In the diagram, HMR-1 is indicated in black, and cell colors are reduced in intensity after the first panel for better contrast with HMR-1. Note the lack of HMR-1::GFP around the v2V and v3V cells, which do not undergo nuclear migration. (F) Intercalation of v2R in an egl-43 mutant. v2R (blue) remains at the interface between v1 and an int1 cell, a [file pgen.1003772.s003.tif]

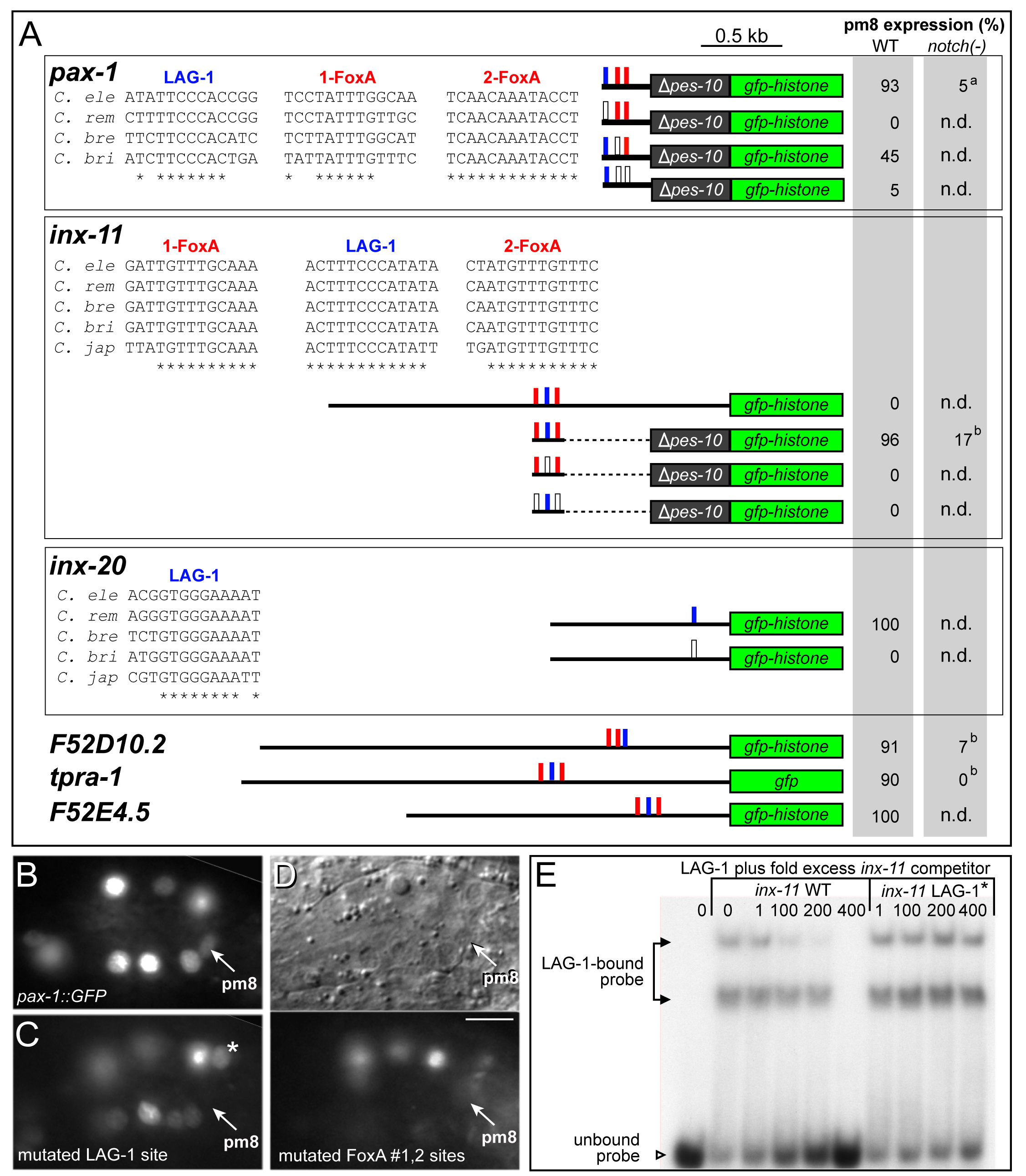

Supplement: Figure S4 — Control of Notch-dependent gene expression in pm8. (A) Diagram of reporter constructs showing conserved LAG-1/CSL and FoxA sequences in orthologous sequences from related nematodes. To test for Notch dependence, pm8 expression was scored in either (a) lin-12(n941) glp-1(q46) or (b) lag-1(q385) embryos as indicated [n = 20–69 for WT; n = 14–98 for notch(-)]. (B–D) pax-1::HIS-GFP expression in the pharynx. (B) Expression of the wild-type pax-1 transgene as in Figure 9B. (C) Expression after mutating the candidate LAG-1 binding site (GTGGGAA) to GAGGCAA; expression persists in the marginal cells, but is absent from pm8. The dorsal nucleus indicated by an asterisk is v1. (D) Differential interference contrast (upper) and fluorescence (lower) micrographs of an embryo expressing a pax-1 transgene in which candidate FoxA sites 1 and 2 have been mutated to TATATGG and TATATGT, respectively. (E) EMSA using a previously described probe that contains two LAG-1 binding sites [43]. Competitor DNA contains either wild-type sequence from inx-11 or a sequence in which the ATGGGAA site has been mutated to AAGGCAA. Bars: (B–D) 2.5 microns. (TIF) [file pgen.1003772.s004.tif]
