# Supplementary material for: Cell Interactions and Patterned Intercalations Shape and Link Epithelial Tubes in C. elegans
Source: PLoS Genet. 2013 Sep 5;9(9):e1003772. doi: 10.1371/journal.pgen.1003772 (PMC3764189; doi:10.1371/journal.pgen.1003772)
Supplement: Text S1 — Supplemental Materials and Methods. (DOCX) [file pgen.1003772.s006.docx]

**Supplemental Materials and Methods**

**Strains**

Nematodes were maintained under standard conditions [1]. The following mutant alleles and genetic deficiencies were used: LG I: *hmr-1(zu389)* [2], *inx-20(ok681)* (isolated by the International *C. elegans* Gene Knockout Consortium); LG II: *die-1(w34)* [3], *tbx-35(tm1789)* [4], *eff-1(ok1021)* [5], *eff-1(ok1021) aff-1(tm2214)* [6], *dpy-10(e128), unc-4(e120)* [1]*, egl-43(n1079)* [7], *egl-43(zu471)* (this study), *eDf21* [8], *mnDf24*, *mnDf57*, *mnDf59, mnDf60*, *mnDf61,* *mnDf71, mnDf83* [9]*;* LG III: *lin-12(n941) glp-1(q46)* [10]; LG IV: *lag-1(q385)* [10]; LG V: *fog-2(q71) pha-4(q490)* [11], *ceh-51(tm2123)* [12]; LG X: *lin-2(e1309)* [13].

We used the following previously described transgenes: *ccIs4595* [*ceh-24*::GFP] [14], *hyEx167* [*aff-1*::GFP] [6], *ltIs44* [*pie-1*::mCherry::PH(PLC1δ1)] [15], *muIs42* [*mig-13*::MIG-13::GFP] [16], *pxls2* [*pax-1*::GFP::HIS-11] [17], *pxIs10* [*pha-4*::GFP::CAAX] [18], *sEx10143* [*F16D3.7*::gfp], *sEx14294* [*F52D10.2*::GFP], *sEx15438* [*K08A8.2a*::GFP] [19], *thEx10* [*tpra-1*::GFP] [20], *wgIs37* [PHA-4::TY1::EGFP::3xFLAG] [21], *xnIs17* [*dlg-1*::DLG-1::GFP] [22], *xnIs96* [*hmr-1*::HMR-1::GFP] [23], *zuIs45* [*nmy-2*::NMY-2::GFP] [24], *zuIs70* [*end-1*::GFP::CAAX] [25], *zuIs104* [*ref-1*^1.8kb^::REF-1::GFP] [26], *zuIs190* [*myo-2*::GFP::HIS-11] [27], *zuEx221* [*ref-1*^153bp^::GFP::CAAX] [27], *zzIs22* [*eff-1*::EFF-1::GFP] [28], *pie-1*::mCherry::PAR-6 [29].

**Transgene Construction**

Standard methods were used to manipulate DNA and create transgenic strains [30]. Mutagenesis of enhancer sequences was performed using the QuikChange II XL Site-Directed Mutagenesis Kit (Stratagene). *zuIs231* and *zuIs232* were isolated following gamma-irradiation of *zuEx236*. Details on the DNA constructs created for this study are listed below. Most constructs used pAP.10 (MCS-*pes-10*::GFP::HIS-11; gift from Jeb Gaudet) [31] or pKG61 (MCS-GFP::HIS-11), which lacks the minimal *pes-10* promoter sequence, as a backbone.

| **Plasmid** | **Description** | **Details** |
| --- | --- | --- |
| pJR09 | *lin-12*^pm8^::*pes-10*::GFP::HIS-11 | A 1.4 kb intronic region of *lin-12* was PCR amplified from N2 genomic DNA with the primers CATGCATGCGGCACTTTTAAGACACCCAAC, CTAGCTAGCGGGACACACGCAAATGTATG  and cloned into pAP.10 (SphI/NheI) |
| pJR11 | *inx-11*::GFP::HIS-11 | A 2.5 kb upstream region of the *W04D2.3a* transcript was PCR amplified from N2 genomic DNA with the primers CTACTGCAGTGTTCACCCGTCAAGTACCA, CGTCGTCGACTGACTGAAAATTTGAGAGGATTGA and cloned into pKG61 (PstI/SalI). |
| pJR14 | *R05G6.10*::GFP::HIS-11 | A 3.0 kb upstream region of the *R05G6.10* transcript was PCR amplified from N2 genomic DNA with the primers CTAGCATGCTGGGACCAGTTTCAACCATT, CGTCTGGCCACCTGTTTGAGACTGCGAAGA and cloned into pKG61 (SphI/MscI). |
| pJR15 | *Y47A7.2*::GFP::HIS-11 | A 2.8 kb upstream region of the *Y47A7.2* transcript was PCR amplified from N2 genomic DNA with the primers CTACTGCAGGCTAAGCTCCCGCCTAAGTT, CGTCGTCGACGGAATGTGTTGCCTGGAAAA and cloned into pKG61 (PstI/SalI). |
| pJR17 | *lin-12*^pm8^::*pes-10*::mCherry::CAAX | mCherry was PCR amplified from pZZ1 (gift of Zhongying Zhao) [32] with the primers GTACCGAGCTCAGAAAAAATGGTCTCAAAGGGTGAAGAAG, GCAGTGTCGTTGCGAATTCATGATCAAGAGGCGCCCTTATACAATTCATCCATGCCACCT  and cloned in place of the GFP::HIS-11 portion of pJR09 (SacI/EcoRI). The CAAX tag was added by annealing the oligos  GCGCATCTCATCGTGAGCGTCACGACAATAATAAGCCACAAAAGAAGAAGAAGTGTCAAATAATGT, GATCACATTATTTGACACTTCTTCTTCTTTTGTGGCTTATTATTGTCGTGACGCTCACGATGAGAT  and ligation (KasI/BclI). |
| pJR18 | *inx-11*^200bp^::*pes-10*::GFP::HIS-11 | A 200 bp fragment of the *inx-11* upstream region was PCR amplified from pJR11 with the primers  CTAGCATGCTCTCTCATCTTATTTTGTTGTTGTGTC, CGTCGTCGACTGCATTTTCATTCTTTCATATTTTG  and cloned into pAP.10 (SphI/SalI). |
| pJR21 | *F52E4.5*::GFP::HIS-11 | A 2.0 kb fragment of upstream of the *F52E4.5* transcript was PCR amplified from N2 genomic DNA with the primers  CTAGCATGCTGGCATTCTACATGCAAAATATG, CTACTGCAGGATCAACTGAAAACATACATTTTAAATCA  and cloned into pKG61 (SphI/PstI). |
| pJR31 | *inx-20*::GFP::HIS-11 | A 1.1 kb fragment of upstream of the *T23H4.1a* transcript was PCR amplified from N2 genomic DNA with the primers  CTACTGCAGTTTTTGTGGCTTCAATTTGG, CGTCGTCGACCGGTGGAGGGGGAAAACAGAA  and cloned into pKG61 (PstI/SalI). |
| pJR45 | *egl-43*::GFP::CAAX | A 8.0 kb fragment of upstream of the *R53.3a* transcript was PCR amplified from fosmid WRM0622cG11 with the primers CGTCCTAAAGCTTCTGGCACACAAAACTCTGATTTA, CGTCGGATCCTTAGGAAACTGTACGTGGGAAGA  and cloned into pKG79 [*ref-1^153bp^*::GFP::CAAX] (HindIII/BamHI) [27]. |
| fJR1 | *egl-43*::EGL-43::GFP fosmid | GFP was recombined in place of the *egl-43* stop codon in fosmid WRM0622cG11 as described [33]. |
| pSSR04 | *lin-12*^pm8^::*pes-10*::GFP::dMoeABD | The dMoeABD domain was PCR amplified from pJWZ6 [34] with primers  CTAGCGGCCGCTCTAGAACTAGTGACGAAGTGGAAGACGCC, CGTCGAATTCATCGTTCACTTTTCACGGATCT  and cloned into pJR09 (NotI/EcoRI). |
| pSSR05 | *lin-12*^pm8^::*pes-10*::mCherry::PH-PLCδ1 | The PH-PLCδ1 domain was PCR amplified from pAA173 [15] with primers  CTAAAGGCGGACCATTACCA, CGTCGAATTCTTACTTCTGCCGCTGGTC and cloned into pJR17 (MluI/EcoRI). |
| pJF32 | *end-1*::mCherry | The *end-1* promoter was PCR amplified from pJN152 [25] using primers  GGCGCGCCACATCGATTTAAACGCCATT, GGCCGGCCATTGTAGATAACAAATGAG  and cloned into pJF25 (Asc1/Fse1). |
| pJF51 | *lin-12*^pm8^::*pes-10*::SAS-5::mCherry | A 1.4 kb intronic region of *lin-12* and the minimal *pes-10* promoter were PCR amplified from pJR09 with the primers  CGTAGGCGCGCCGCGGCACTTTTAAGACACC, CATGGGCCGGCCTTTTTCTGAGCTCGGTACCCTCCA  and cloned into pJF31 (Asc1/Fse1). |
| pJF53 | *lin-12*^pm8^::*pes-10*::PAR-6::GFP | The *lin-12* enhancer and *pes-10* promoter were cloned into pJF43 (Asc1/Fse1). |
| pKG68 | *pax-1*::*pes-10*::GFP::HIS-11 | A 298 bp fragment upstream of *pax-1* was PCR amplified from N2 genomic DNA with the primers  ATACCCTGCAGCAGATGTTAAAACACACCCGT, ATTAGCTGCAGGCTCGAGCGAGTGTGACTG and cloned into pAP.10 (PstI). |

Unstable extrachromosal transgenes created for this study:

| **Extrachromosomal array(s)** | **Constructs** |
| --- | --- |
| *zuEx236, zuEx237* | pJR09, pRF4 |
| *zuEx254* | pJR09, pJR17, pRF4 |
| *zuEx240, zuEx244* | pJR11, pRF4 |
| *zuEx248, zuEx249* | pJR14, pRF4 |
| *zuEx250, zuEx251* | pJR15, pRF4 |
| *zuEx252, zuEx253* | pJR18, pRF4 |
| *zuEx256, zuEx257* | pJR31, pRF4 |
| *zuEx270* | pJR45, pRF4 |
| *zuEx267, zuEx268, zuEx269* | fJR1, pRF4 |
| *zuEx290, zuEx291* | fJR1, pJF32, pCFJ90 [35] |
| *zuEx271* | pSSR04, pSSR05, pRF4 |
| *zuEx299* | pJF51, pJF53, pRF4 |
| *zuEx145* | pKG68, pRF4 |

**Isolation and Mapping of *zu471***

*zuIs190* [*myo-2*::GFP::HIS-11] *II; lin-2(e1309) X* L4 hermaphrodites were mutagenized with ethyl methanesulfonate and their F2 progeny screened for pharyngeal defects as described [2]. From this screen we recovered the L1 lethal mutants *zu470* and *zu471*. *zu471* was mapped to LG II based on its linkage to *zuIs190*. Three factor mapping against *dpy-10(e128) unc-4(e120)* revealed that *zu471* is likely to the right of *unc-4*. Specifically, all 10 of the Dpy non-Unc recombinants picked up *zu471* whereas none of the 4 Unc non-Dpy recombinants picked up *zu471*. To further refine the position of *zu471* we used deficiency mapping. We found that *zu471* complemented the deficiencies *mnDf60*, *mnDf61*, and *mnDf83*, but failed to complement the deficiencies *eDf21*, *mnDf24*, *mnDf57*, *mnDf59* and *mnDf71*. This placed *zu471* in the ~0.15 Mb interval between *unc-4* and *flp-4*. On the basis of this map position, we tested whether *zu471* was a new allele of *egl-43*. We found that *zu471* failed to complement the Egl phenotype of the partial loss-of-function allele *egl-43(n1079)*. We sequenced all *egl-43* exons from animals homozygous for *zu471* and identified the single missense mutation R489>H. *zu471* was outcrossed eight times and maintained over *mnC1[dpy-10(e128) unc-52(e444)]* [36] or *mIn1[dpy-10(e128) mIs14]* [37].

**DNA Binding**

Electrophoretic mobility shift assays were performed as described [38]. The wild-type *inx-11* probe was: GACAAGATTGTTTGCAAATCATCACTTTCCCATATACTTTTCAATGCCTTTCTATGTTTGTTTCGCTCC.

1. Brenner S (1974) The genetics of Caenorhabditis elegans. Genetics 77: 71-94.

2. Costa M, Raich W, Agbunag C, Leung B, Hardin J, et al. (1998) A putative catenin-cadherin system mediates morphogenesis of the Caenorhabditis elegans embryo. J Cell Biol 141: 297-308.

3. Heid PJ, Raich WB, Smith R, Mohler WA, Simokat K, et al. (2001) The zinc finger protein DIE-1 is required for late events during epithelial cell rearrangement in C. elegans. Dev Biol 236: 165-180.

4. Broitman-Maduro G, Lin KT, Hung WW, Maduro MF (2006) Specification of the C. elegans MS blastomere by the T-box factor TBX-35. Development 133: 3097-3106.

5. Podbilewicz B, Leikina E, Sapir A, Valansi C, Suissa M, et al. (2006) The C. elegans developmental fusogen EFF-1 mediates homotypic fusion in heterologous cells and in vivo. Dev Cell 11: 471-481.

6. Sapir A, Choi J, Leikina E, Avinoam O, Valansi C, et al. (2007) AFF-1, a FOS-1-regulated fusogen, mediates fusion of the anchor cell in C. elegans. Dev Cell 12: 683-698.

7. Desai C, Horvitz HR (1989) Caenorhabditis elegans mutants defective in the functioning of the motor neurons responsible for egg laying. Genetics 121: 703-721.

8. Shen MM, Hodgkin J (1988) mab-3, a gene required for sex-specific yolk protein expression and a male-specific lineage in C. elegans. Cell 54: 1019-1031.

9. Sigurdson D, Spanier G, Herman R (1984) Caenorhabditis elegans deficiency mapping. Genetics 108: 331-345.

10. Lambie EJ, Kimble J (1991) Two homologous regulatory genes, lin-12 and glp-1, have overlapping functions. Development 112: 231-240.

11. Mango SE, Lambie EJ, Kimble J (1994) The pha-4 gene is required to generate the pharyngeal primordium of Caenorhabditis elegans. Development 120: 3019-3031.

12. Broitman-Maduro G, Owraghi M, Hung WW, Kuntz S, Sternberg PW, et al. (2009) The NK-2 class homeodomain factor CEH-51 and the T-box factor TBX-35 have overlapping function in C. elegans mesoderm development. Development 136: 2735-2746.

13. Horvitz HR, Sulston JE (1980) Isolation and genetic characterization of cell-lineage mutants of the nematode Caenorhabditis elegans. Genetics 96: 435-454.

14. Harfe BD, Fire A (1998) Muscle and nerve-specific regulation of a novel NK-2 class homeodomain factor in Caenorhabditis elegans. Development 125: 421-429.

15. Kachur TM, Audhya A, Pilgrim DB (2008) UNC-45 is required for NMY-2 contractile function in early embryonic polarity establishment and germline cellularization in C. elegans. Dev Biol 314: 287-299.

16. Sym M, Robinson N, Kenyon C (1999) MIG-13 positions migrating cells along the anteroposterior body axis of C. elegans. Cell 98: 25-36.

17. Fakhouri TH, Stevenson J, Chisholm AD, Mango SE (2010) Dynamic chromatin organization during foregut development mediated by the organ selector gene PHA-4/FoxA. PLoS Genet 6.

18. Portereiko MF, Mango SE (2001) Early morphogenesis of the Caenorhabditis elegans pharynx. Dev Biol 233: 482-494.

19. Hunt-Newbury R, Viveiros R, Johnsen R, Mah A, Anastas D, et al. (2007) High-throughput in vivo analysis of gene expression in Caenorhabditis elegans. PLoS Biol 5: e237.

20. Wang P, Zhao J, Corsi AK (2006) Identification of novel target genes of CeTwist and CeE/DA. Dev Biol 293: 486-498.

21. Zhong M, Niu W, Lu ZJ, Sarov M, Murray JI, et al. (2010) Genome-wide identification of binding sites defines distinct functions for Caenorhabditis elegans PHA-4/FOXA in development and environmental response. PLoS Genet 6: e1000848.

22. Totong R, Achilleos A, Nance J (2007) PAR-6 is required for junction formation but not apicobasal polarization in C. elegans embryonic epithelial cells. Development 134: 1259-1268.

23. Achilleos A, Wehman AM, Nance J (2010) PAR-3 mediates the initial clustering and apical localization of junction and polarity proteins during C. elegans intestinal epithelial cell polarization. Development 137: 1833-1842.

24. Munro E, Nance J, Priess JR (2004) Cortical flows powered by asymmetrical contraction transport PAR proteins to establish and maintain anterior-posterior polarity in the early C. elegans embryo. Dev Cell 7: 413-424.

25. Wehman AM, Poggioli C, Schweinsberg P, Grant BD, Nance J (2011) The P4-ATPase TAT-5 inhibits the budding of extracellular vesicles in C. elegans embryos. Curr Biol 21: 1951-1959.

26. Neves A, Priess JR (2005) The REF-1 family of bHLH transcription factors pattern C. elegans embryos through Notch-dependent and Notch-independent pathways. Dev Cell 8: 867-879.

27. Rasmussen J, English K, Tenlen J, Priess J (2008) Notch signaling and morphogenesis of single-cell tubes in the C. elegans digestive tract. Dev Cell 14: 559-569.

28. del Campo JJ, Opoku-Serebuoh E, Isaacson AB, Scranton VL, Tucker M, et al. (2005) Fusogenic activity of EFF-1 is regulated via dynamic localization in fusing somatic cells of C. elegans. Curr Biol 15: 413-423.

29. Schonegg S, Constantinescu AT, Hoege C, Hyman AA (2007) The Rho GTPase-activating proteins RGA-3 and RGA-4 are required to set the initial size of PAR domains in Caenorhabditis elegans one-cell embryos. Proc Natl Acad Sci U S A 104: 14976-14981.

30. Mello C, Fire A (1995) DNA transformation. Methods Cell Biol 48: 451-482.

31. Gaudet J, Mango SE (2002) Regulation of organogenesis by the Caenorhabditis elegans FoxA protein PHA-4. Science 295: 821-825.

32. Zhao Z, Boyle TJ, Liu Z, Murray JI, Wood WB, et al. (2010) A negative regulatory loop between microRNA and Hox gene controls posterior identities in Caenorhabditis elegans. PLoS Genet 6.

33. Tursun B, Cochella L, Carrera I, Hobert O (2009) A toolkit and robust pipeline for the generation of fosmid-based reporter genes in C. elegans. PLoS One 4: e4625.

34. Ziel J, Hagedorn E, Audhya A, Sherwood D (2009) UNC-6 (netrin) orients the invasive membrane of the anchor cell in C. elegans. Nat Cell Biol 11: 183-189.

35. Frøkjaer-Jensen C, Davis MW, Hopkins CE, Newman BJ, Thummel JM, et al. (2008) Single-copy insertion of transgenes in Caenorhabditis elegans. Nat Genet 40: 1375-1383.

36. Herman RK (1978) Crossover suppressors and balanced recessive lethals in Caenorhabditis elegans. Genetics 88: 49-65.

37. Edgley ML, Riddle DL (2001) LG II balancer chromosomes in Caenorhabditis elegans: mT1(II;III) and the mIn1 set of dominantly and recessively marked inversions. Mol Genet Genomics 266: 385-395.

38. Neves A, English K, Priess JR (2007) Notch-GATA synergy promotes endoderm-specific expression of ref-1 in C. elegans. Development 134: 4459-4468.
